# Assessing the Effect of Explicit Polarizability on Models of Carbon Dioxide Solvation in Ionic Liquids

**DOI:** 10.1021/acsomega.5c08258

**Published:** 2025-11-11

**Authors:** Zijian Huo, Logan E. Smith, Rowan J. Goudy, Khady Ndiaye, Scott Kaiser, Marie E. Nikolov, Ethan Silva, Tyler A. Parrack, Sean Garrett-Roe, Clyde A. Daly

**Affiliations:** † Department of Chemistry, 3776 Haverford College, 370 Lancaster Avenue, Haverford, Pennsylvania 19041, United States; ‡ Department of Chemistry, Bryn Mawr College, 101 N Merion Avenue, Bryn Mawr, Pennsylvania 19010, United States; § Department of Chemistry, 6614 University of Pittsburgh, 219 Parkman Avenue, Pittsburgh, Pennsylvania 15260, United States

## Abstract

Ionic liquids are an important possible carbon capture material because of their anomalously high sorption selectivity for carbon dioxide over other gases common in air. Many research groups have investigated the molecular origins of this property and provided important insights, including using 1D and 2D-IR spectroscopy. Molecular dynamics simulations have been indispensable to the interpretation of these experiments. In prior molecular dynamics simulation work, charge-scaled force fields have typically been used to provide a mean-field treatment of effects vital to ionic liquid systems such as charge transfer and polarization. Here, we compare models of carbon dioxide solvated in ionic liquids with explicit polarization to models of the same with implicit polarizability through charge-scaling. We calculate structural, dynamical, and spectroscopic properties, and make comparisons to the same items measured in experiment. In this study, we focus on two ionic liquids: 1-butyl-3-methylimidazolium (BMIM^+^) paired with bis­(trifluoromethane sulfonyl imide) (Tf_2_N^–^) and 1-butyl-3-methylimidazolium (BMIM^+^) paired with hexafluorophosphate (PF_6_^–^). We find that many structural, dynamical, and spectroscopic properties are changed when polarization is modeled explicitly. We also find that explicit polarizability softens local ion cages around the carbon dioxide and that the long-time diffusion of the carbon dioxide is gated by the reorganization of the ionic liquid molecules. Comparisons to experiment show modest improvement of many observables compared with experiment for the explicitly polarizable model over the charge-scaled model. Overall, our results show that charge-scaled force fields are likely sufficient to compute spectroscopic properties of carbon dioxide in ionic liquids and suggest some interpretive rules for understanding their structural and dynamical properties. Those using charge-scaled force fields should generally assume that the ion cages around solutes such as carbon dioxide are too stiff and cation-rich in their models and adjust their interpretations and predictions accordingly.

## Graphical abstract

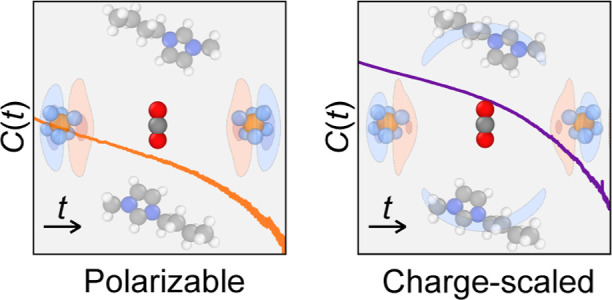

## Introduction

The rapid rise of carbon dioxide (CO_2_) levels in the atmosphere has accelerated global warming in recent decades, driving severe climate change and extreme weather conditions.^1–4^ To address these effects, it is crucial not only to reduce CO_2_ emissions but also to remove existing atmospheric CO_2_ through carbon capture materials, including amines,^5^ metal–organic frameworks,^6^ and polymer membranes.^7^ In addition to these materials, ionic liquids (ILs) have also emerged as a promising candidate for carbon capture.^8^ ILs are salts that remain in the liquid state at or near room temperature. These materials have been widely investigated as a potential absorbent for carbon capture applications due to their unique properties, including low vapor pressure, high thermal and chemical stability, and strong CO_2_ solubility.^9,10^ IL sorption capacity depends strongly on the interactions between CO_2_ molecules and the ions, and these interactions have proven to be especially important in understanding and predicting CO_2_ solubility.^9,11,12^ The CO_2_ molecule has been shown to form long-lived ion cages due to its substantial quadrupole moment, and the anions are an integral part of these cages.^13,14^ In general, understanding how the solvation environment around the CO_2_ molecule alters its structural properties and dynamic behaviors is critical for designing efficient absorbents in carbon capture applications, regardless of which material is used. Moreover, studies of ILs can also advance our understanding of this specific material for carbon capture applications and suggest design principles for developing the next generation of carbon capture materials.

Atomistic molecular dynamics (MD) simulations have been widely employed as a powerful tool to systematically investigate the molecular interactions between CO_2_ and IL ions, and how these interactions influence the solvation behavior of CO_2_.^15–24^ To accurately simulate these systems, the IL ions and CO_2_ must be described using compatible force fields. These force fields describe the bonding within each molecule and how atoms in different molecules interact with each other.^25^ In early work, IL ions were often modeled using traditional nonpolarizable force fields with fixed atomic charges. It was found that these models overestimated the attractions between ions, leading to unrealistic dynamical properties.^26^ A common and computationally inexpensive approach to correct this is to scale the charges by a factor of ∼0.8 to account for the overall effects of polarization and charge transfer between the ions.^27,28^ The specific scaling factor for a given IL is typically chosen to match experimental thermodynamic properties such as viscosity, heat of vaporization, and diffusion coefficient.^26,28^ When paired with a fixed-charge model of CO_2_, this approach can successfully reproduce both solvent–solvent and solute–solvent interactions and provide reasonable predictions of the structural and dynamical properties of CO_2_ molecules in ILs.^12,13,19,29^ Nonetheless, certain properties remain challenging to capture accurately. For instance, in prior work on CO_2_ in [BMIM^+^]­[PF_6_^–^], the simulated diffusion constant of the CO_2_ was too large by nearly a factor of 2 when compared with the experimental value.^13,30^ It was also found that the full width at half-maximum (fwhm) of the IR spectrum for the asymmetric stretch of CO_2_ was too narrow.^14^ Since this vibrational mode is a useful experimental probe of the CO_2_ structure and dynamics in ILs, understanding the origin of discrepancies between the experimental and theoretical spectra is essential for improving simulation accuracy.^14,31^

To overcome the limitations of nonpolarizable force fields, several teams including McDaniel et al. and Goloviznina et al. have introduced polarizable force fields for ILs that explicitly model the effect of polarization using Drude oscillators.^32,33^ While this approach requires a significant increase in computational cost due to the addition of Drude particles, it provides a promising improvement in accurately describing both the structural and dynamic properties of neat ILs. There has also been some work that extends these polarizable force fields to explore more complex systems, such as ILs in polymer matrices.^34^ However, relatively few studies have focused on examining the effect of polarizable force fields on the interactions between IL ions and solutes besides water.^26^

In this work, we compare the structural, dynamic, and spectroscopic properties of CO_2_ in two well-studied IL systems, shown in Figure 1: 1-butyl-3-methylimidazolium paired with bis­(trifluoromethane sulfonyl imide) ([BMIM^+^]­[Tf_2_N^–^]) and hexafluorophosphate ([BMIM^+^]­[PF_6_^–^]). This comparison allows us to focus on the effects on the solvation of CO_2_ in ILs arising when polarization is modeled explicitly (in the polarizable force field) as opposed to implicitly (in the nonpolarizable charge-scaled force field). Comparing these two ILs also allows us to isolate the effects that are due to explicit polarization from those that are particular to one ionic liquid. We began this study with the expectation that incorporating explicit polarization would substantially improve the predicted IR spectrum of CO_2_ compared to the experimental measurements. We did find that explicit polarizability improves the predicted CO_2_ diffusion constant, the IR spectrum, and some other dynamical properties. However, the improvement is inconsistent and may not justify the additional expense of these force fields for predicting them specifically for the solvated CO_2_ molecule. Explicit polarizability does induce significant changes to a number of structural and dynamical properties for CO_2_ in ILs, which should be kept in mind when assessing the experimental implications of results obtained using a charge-scaled force field.

**Figure 1:**
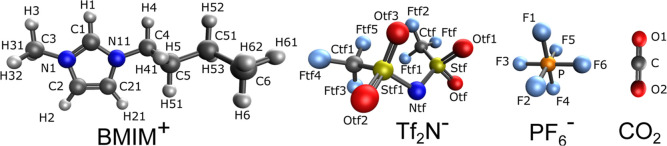
Molecular structure of the molecules studied in this work, with corresponding atom names used in MD simulations.

## Results

### Ion Cage Structure

The solvation structures of CO_2_ in ILs, modeled using polarizable and nonpolarizable force fields, were analyzed using atomistic cylindrical distribution functions (aCDFs) as shown in Figure 2. Both models indicate that the CO_2_ molecule resides within a solvent cage with a specific well-defined structure. The two force fields also agree on some of the details of the solvent cage structure. In both cases, atoms associated with the cation alkyl tails are uniformly distributed around the CO_2_ with relatively low intensity and slight biases for associating with the CO_2_ carbon. Atoms associated with the cation ring are localized at high and low *z*, near the oxygen atoms of the CO_2_ oxygen atoms. Finally, atoms associated with the anions are localized near the CO_2_ carbon, near *z* ≈ 0.

**Figure 2:**
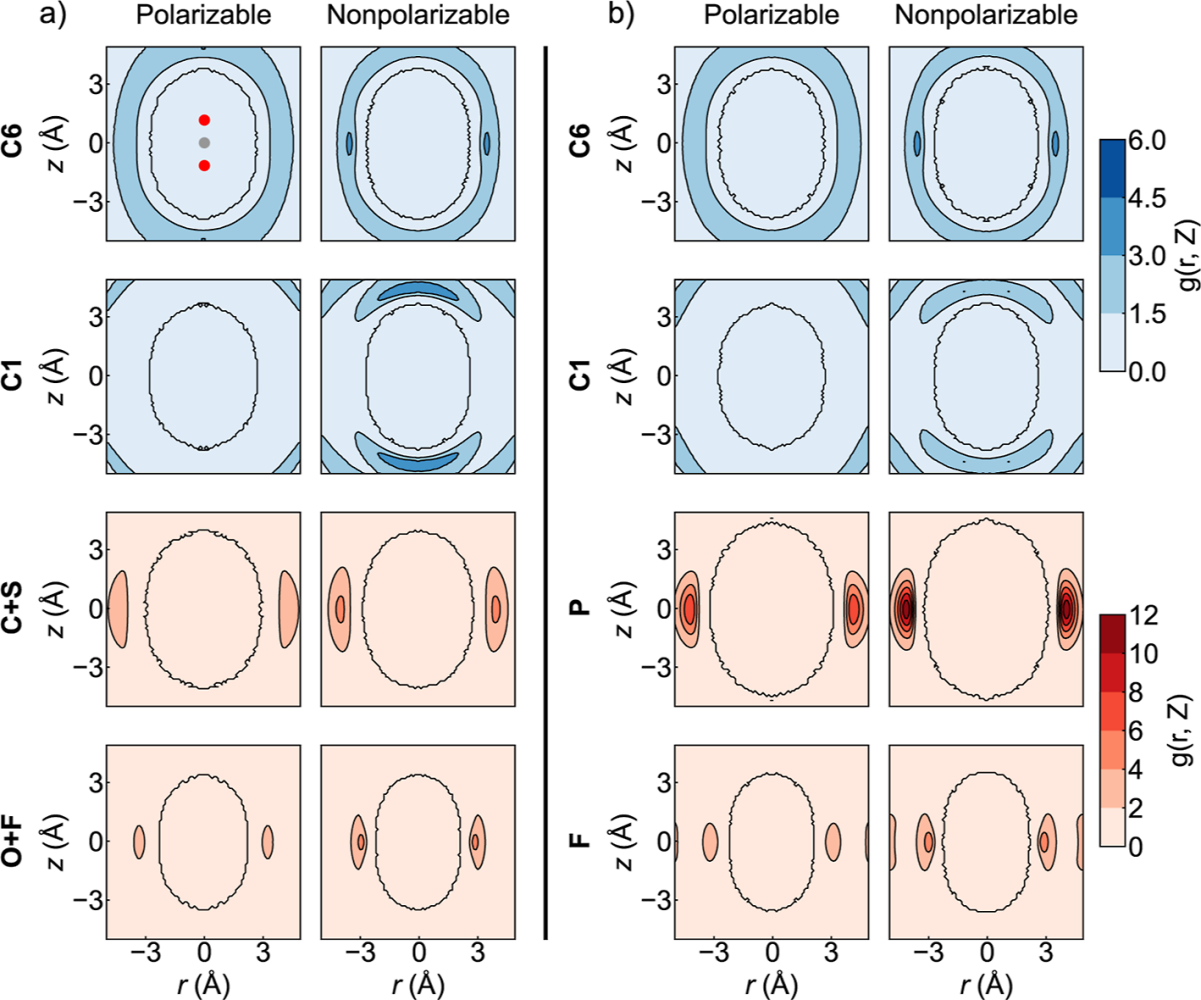
Time-independent atomic density distribution around a single CO_2_ molecule in (a) [BMIM^+^]­[Tf_2_N^–^] and (b) [BMIM^+^]­[PF_6_^–^] with polarizable and nonpolarizable force fields. The CO_2_ molecule is centered in the plot as shown in the first figure, with the gray circle representing the carbon atom and the red circles representing the oxygen atoms. C6 represents the terminal carbon atom of the butyl group in [BMIM^+^]. C1 represents the internitrogen carbon atom in the imidazole ring of [BMIM^+^]. C, S, O, F, and P represent the carbon, sulfur, oxygen, fluorine, and phosphorus atoms in [Tf_2_N^–^] and [PF_6_^–^]. The C and S atoms in [Tf_2_N^–^] are averaged together to represent “central” atoms that do not make direct contact with the CO_2_, and so are best compared to the *P* in [PF_6_^–^]. The O and F in [Tf_2_N^–^] are compared to the F in [PF_6_^–^] in a similar way. Each plot represents an average over all possible time separations of the appropriate length over all ten 100 ns simulations. Plots of the relative 95% confidence intervals across the ten 100 ns simulations are given in the Supporting Information.

There are also significant differences between the two force fields. In both explicitly polarizable cases, the density of anion atoms is slightly lower, and the cation rings are further away from the CO_2_ oxygens than in the nonpolarizable cases. Between the two ILs, we find that the anion atom densities are higher for [BMIM^+^]­[PF_6_^–^] than those for [BMIM^+^]­[Tf_2_N^–^]. Counting the ions in the first solvation shell over the course of the simulation (Table 1) confirms that there are fewer anions and cation rings near the CO_2_ when polarizability is explicitly modeled; instead, the CO_2_ spends more time near the less charged cation alkyl tails. However, the total number of ion moieties in the first solvation shell does not change much under explicit polarization, especially in [BMIM^+^]­[PF_6_^–^].

**Table 1:** Average Ion Counts in the First Solvation Shell (|*r*| ≤ 5 Å and |*z*| ≤ 5 Å) for the Anion, the Cation Ring, and the Cation Tail for the CO_2_ in [BMIM^+^]­[Tf_2_N^–^] and [BMIM^+^]­[PF_6_^–^]­^a^

system	[BMIM^+^][Tf_2_N^–^]		[BMIM^+^][PF_6_^–^]	
	pol	nonpol	pol	nonpol
anion	1.45 ± 0.01	1.78 ± 0.01	2.15 ± 0.02	2.40 ± 0.02
cation ring	0.97 ± 0.01	1.55 ± 0.01	1.83 ± 0.02	2.07 ± 0.02
cation tail	1.93 ± 0.03	1.81 ± 0.02	2.87 ± 0.04	2.30 ± 0.03
total	4.35 ± 0.02	5.14 ± 0.01	6.85 ± 0.03	6.77 ± 0.02

a For [Tf_2_N^–^], the C and S atoms were used, weighted by the number of C and S atoms in a single [Tf_2_N^–^] anion, and for [PF_6_^–^], the P atom was used as the representative atom to determine the ion counts. For the cation ring, the internitrogen carbon atom (C1) located in the [BMIM^+^] ring was used. For the cation tail, the last carbon atom (C6) located in the [BMIM^+^] butyl group was used. Entries are mean values collected from ten 100 ns simulations, and error bars represent the 95% confidence intervals.

### Ion Cage Dynamics

While the aCDFs provide an effective snapshot of particular moieties around the CO_2_, charge-based cylindrical distribution functions (qCDFs) can offer a broader picture of the solvation shell structure. Time-dependent charge cylindrical distribution functions (*t*-qCDFs) provide a picture of solvation dynamics as well. *t*-qCDFs are shown in Figure 3. In Figure 3a, the charge density in both ILs is found to be largely unchanged between 0 and 1 ps. This implies that the CO_2_ molecule is tightly locked in place within the solvent cage during the first 1 ps of the simulation. During the period between 1 and 100 ps, the charge density of the solvation cage gradually decays. By 1000 ps, the solvent cage had almost completely disappeared in all cases, implying that the CO_2_ molecule has migrated to a new solvent cage within the 100–1000 ps time frame. The polarizable model of [BMIM^+^]­[PF_6_^–^] shows somewhat distinct behavior. Even after 1000 ps, a significant amount of charge density from the initial cage is still present. This suggests that some CO_2_ molecules remain trapped in the initial solvent cage even after 1000 ps when modeled with the polarizable force field, specifically for the [BMIM^+^]­[PF_6_^–^] solvent.

**Figure 3:**
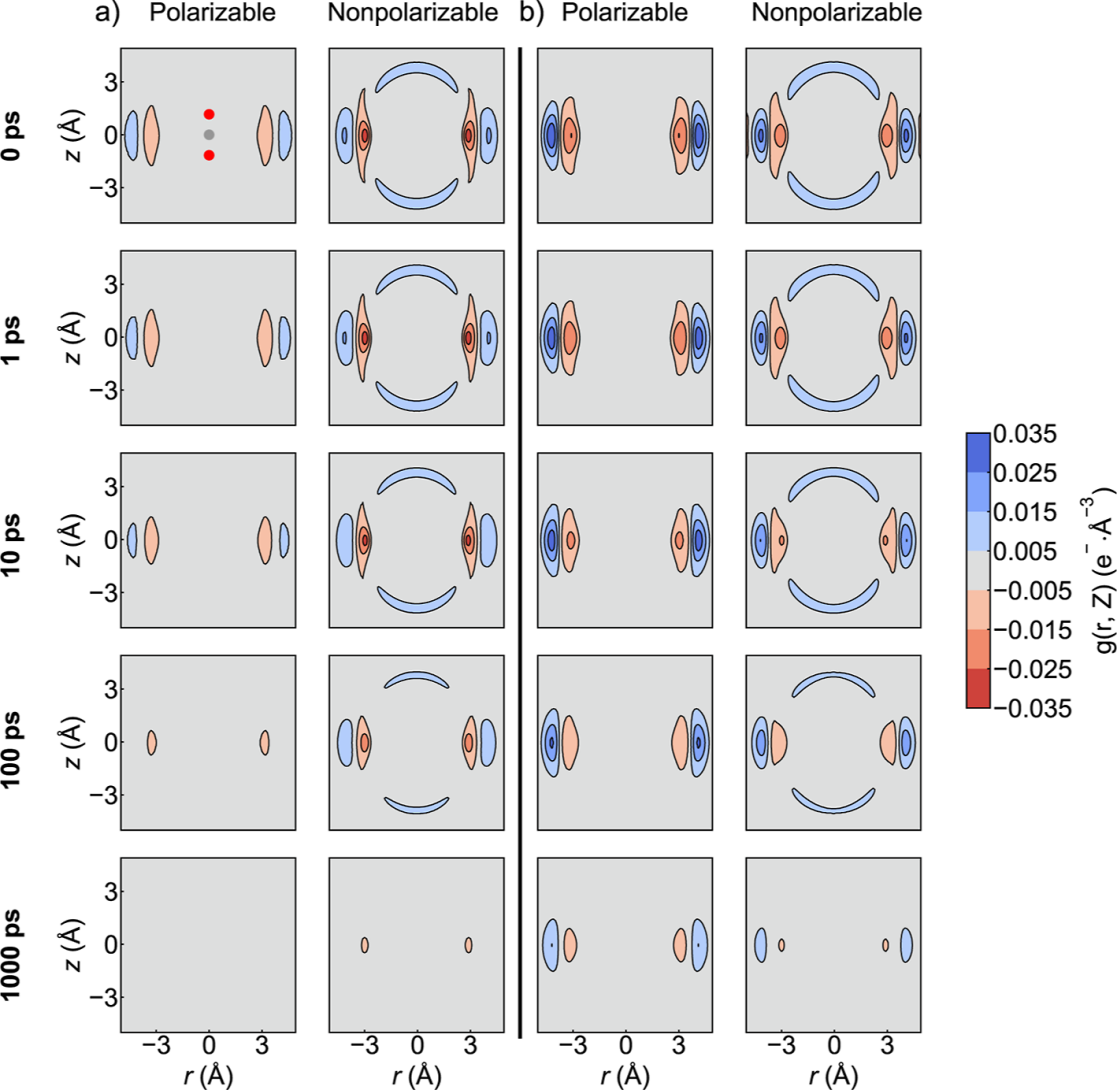
Charge density of (a) [BMIM^+^]­[Tf_2_N^–^] and (b) [BMIM^+^]­[PF_6_^–^] around a single CO_2_ molecule with polarizable and nonpolarizable force fields over time. Each plot represents an average over all possible time separations of the appropriate length over all ten 100 ns simulations. Plots of the relative 95% confidence intervals across the ten 100 ns simulations are given in the Supporting Information.

Clear and consistent differences were also observed between the solvent cages. As seen in the aCDFs, the solvent cages observed using nonpolarizable models have a positive (cation) charge density associated with the CO_2_ oxygen atoms and a negative (anion) charge density associated with the CO_2_ carbon. The polarizable models instead show no significant cation density near the CO_2_ oxygen atoms. Instead, the cage seems to be made completely from the anions surrounding the CO_2_ carbon. Combined with the data from Table 1, this implies that interactions between the CO_2_ and cations are transitory in the polarizable model. Here, the CO_2_ molecule has much longer-lived and more orientationally specific interactions with the anions compared to the nonpolarizable model.

### CO_2_ Translational Diffusion

The *t*-qCDFs suggest that the CO_2_ molecule moves between local ion cages more rapidly in [BMIM^+^]­[Tf_2_N^–^], and our diffusion analysis further supports this finding. Mean square displacements (MSDs) were computed based on simulations containing 5 CO_2_ molecules and 256 ion pairs. For each CO_2_, the MSD was computed and then averaged across the five solute molecules at each time separation, producing Figure 4a,b. Similarly, for each solvent ion, the MSD was computed and averaged over all 256 ions, with the results provided in the Supporting Information. The diffusion coefficients were then obtained from the slope of the MSD curves for each CO_2_ and ion molecule over the 0.4–4.0 ns time interval. In Table 2, the average diffusion constants across each set of 5 CO_2_ molecules and 256 ions are reported along with 95% confidence intervals.

**Figure 4:**
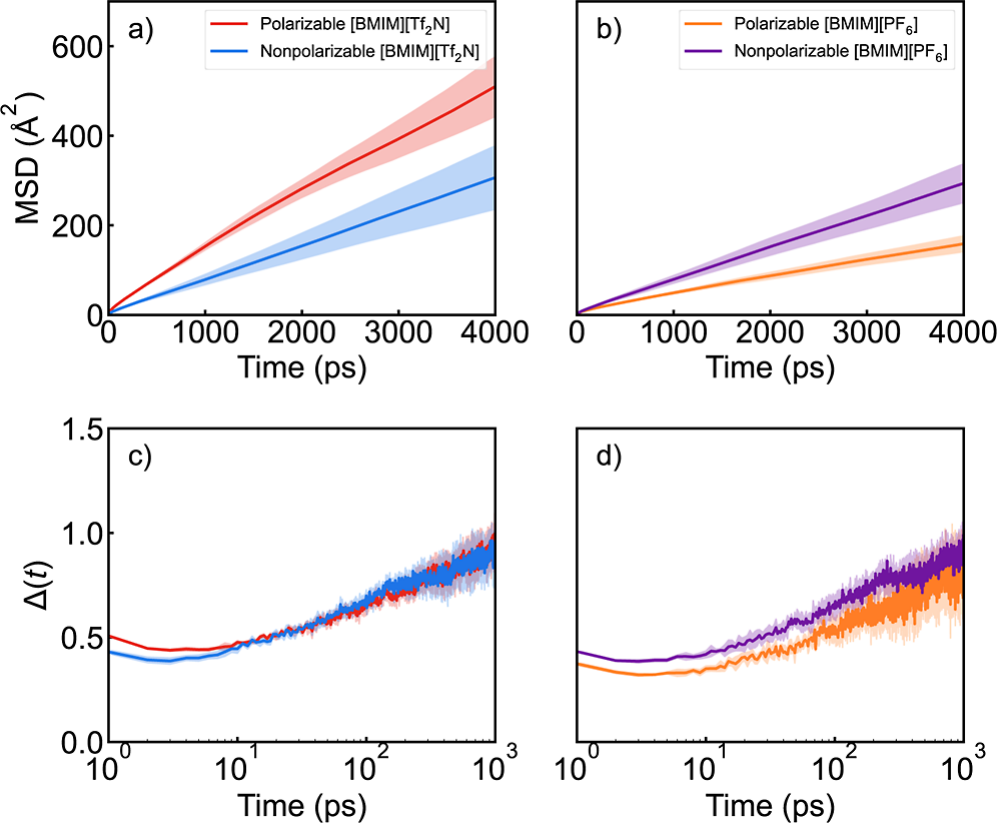
MSD of CO_2_ between 0 and 4 ns in (a) [BMIM^+^]­[Tf_2_N^–^] and (b) [BMIM^+^]­[PF_6_^–^] with polarizable and nonpolarizable force fields. CO_2_ Localization in (c) [BMIM^+^]­[Tf_2_N^–^] and (d) [BMIM^+^]­[PF_6_^–^] with polarizable and nonpolarizable force fields. The shaded region represents the 95% confidence interval of the collected data.

**Table 2:** Diffusion Coefficient of CO_2_ and Ion Molecules at 300 K and 1 bar in [BMIM^+^]­[Tf_2_N^–^] and [BMIM^+^]­[PF_6_^–^]

value	[BMIM^+^][Tf_2_N^–^]		[BMIM^+^][PF_6_^–^]	
	pol	nonpol	pol	nonpol
*D̵*_CO_2__ (Å^2^/ns)	20.0 ± 3.3	12.6 ± 3.3	6.2 ± 0.9	11.9 ± 1.9
experimental^35,36^ (Å^2^/ns)	8.5 ± 1.9		5.7 ± 0.1	
*D̵*_+_ (Å^2^/ns)	1.37 ± 0.13	0.63 ± 0.05	0.25 ± 0.03	0.44 ± 0.05
experimental^37,38^ (Å^2^/ns)^a^	2.75 ± 0.42		0.69 ± 0.02	
*D̵*_–_ (Å^2^/ns)	0.85 ± 0.08	0.55 ± 0.06	0.16 ± 0.02	0.27 ± 0.03
experimental^37,38^ (Å^2^/ns)^a^	2.10 ± 0.33		0.52 ± 0.02	

a The reported values are from neat ILs.

The calculated diffusion coefficients confirm that the CO_2_ molecule exhibits faster diffusion in [BMIM^+^]­[Tf_2_N^–^] than in [BMIM^+^]­[PF_6_^–^] with the same type of force field. In [BMIM^+^]­[PF_6_^–^], diffusion of CO_2_ is faster in the nonpolarizable model than in the polarizable model. Explicit polarizability also tends to slow the motion of the ions in this liquid. The reverse is observed for [BMIM^+^]­[Tf_2_N^–^]—explicit polarizability accelerates the diffusion of CO_2_ and of the ions. Faster diffusion and other dynamical properties are a commonly observed effect of explicit polarizability, but the slowdown observed here for [BMIM^+^]­[PF_6_^–^] has been observed elsewhere.^33,51,77^ For both models and both ILs, faster diffusion of CO_2_ is correlated with faster diffusion of the ions.

Although the diffusion constants obtained from the polarizable model generally show better agreement when compared to the experimental values, we find that for [BMIM^+^]­[Tf_2_N^–^], the diffusion constants for the CO_2_ agree moderately better with the nonpolarizable model. In contrast, the polarizable force field shows better agreement with the experimental results for the [BMIM^+^]­[PF_6_^–^] system.

The scaling of the MSD with time separation, *t*, can be used to better understand how the solvent cage affects CO_2_ dynamics. Here, we use the function

1 $$\Delta \left(t\right)\equiv \frac{\partial \log\left(MSD\right)}{\partial \log\left(\tau \right)}$$

to extract the scaling (Figure 4c,d).^39^ At *t* = 0, the particle is expected to be in the ballistic regime so that Δ­(*t*) = 2. As *t* → ∞, Δ­(*t*) → 1 as the particle undergoes random diffusion. The ballistic regime is complete in femtoseconds (data not shown), and we see that Δ­(*t*) correctly tends toward 1 at long times. Between 1 and 5 ps, there is a minimum in Δ­(*t*). This is expected and represents collisions between the CO_2_ and ions within its local cage. The time at which this minimum occurs, t*, represents the average time it takes for these collisions to take place. Plugging t* back into MSD provides an estimate of the “caging area” available to the CO_2_ in a given solvent cage, MSD­(t*).

To extract t*, Δ­(*t*) data from 1 to 5 ps were fit to a quadratic function, i.e., Δ­(*t*) = *at* ^2^ + *bt* + *c*. The time at which this function was minimized is taken as t*. t* was used to estimate the caging area, MSD­(t*), by fitting MSD data to the equation $MSD\left(t\right)={mt}^{\Delta \left(t^{*}\right)}$ in the 1 to 5 ps range. The resulting data are listed in Table 3.

**Table 3:** Cage Collision Times and Caging Areas of CO_2_ in [BMIM^+^]­[Tf_2_N^–^] and [BMIM^+^]­[PF_6_^–^]

system	[BMIM^+^][Tf_2_N^–^]		[BMIM^+^][PF_6_^–^]	
	pol	nonpol	pol	nonpol
t*(ps)	4.6 ± 0.3	3.4 ± 0.2	4.5 ± 0.4	3.9 ± 0.4
MSD(*t* ^*^)(Å^2^)	5.0 ± 0.2	2.3 ± 0.1	3.0 ± 0.1	2.7 ± 0.2

Table 3 shows that the cage collision time is about 1 ps longer for polarizable force fields, resulting from larger cages. The larger apparent cage size is most likely due to the overall softening of the ion cages in the polarizable case, as seen in all CDFs. The cage collision time and caging areas of CO_2_ are similar in both [BMIM^+^]­[Tf_2_N^–^] and [BMIM^+^]­[PF_6_^–^] with nonpolarizable force fields. Under the polarizable force fields, the cage collision time is nearly identical for both ILs. However, the caging area is significantly larger in [BMIM^+^]­[Tf_2_N^–^], which may result in faster CO_2_ dynamics in the polarizable [BMIM^+^]­[Tf_2_N^–^] system.

### CO_2_ Orientational Diffusion

Another important way to quantify the dynamic behavior of a molecule is through its orientational diffusion, captured through orientational correlation functions (OCFs) (eq 11). These correlation functions are listed in Figure 5.

**Figure 5:**
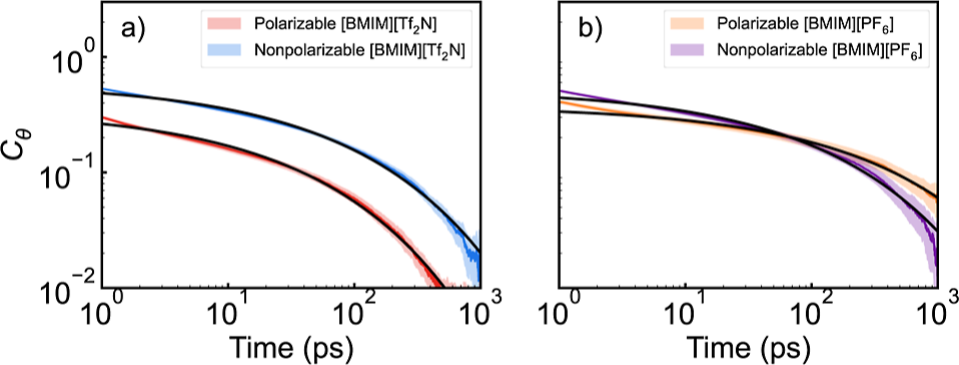
OCFs of CO_2_ between 1 and 1000 ps in (a) [BMIM^+^]­[Tf_2_N^–^] and (b) [BMIM^+^]­[PF_6_^–^] with polarizable and nonpolarizable force fields. The shaded region represents the 95% confidence interval of the collected data across ten 100 ns simulations. Fitted curves are shown in black.

At short time scales, the rotational memory of the CO_2_ molecule decays less rapidly in the systems modeled with nonpolarizable force fields compared to those modeled with polarizable force fields. However, the OCFs exhibit a “switch” at longer times in [BMIM^+^]­[PF_6_^–^] but not in [BMIM^+^]­[Tf_2_N^–^]. This indicates that the nonpolarizable model leads to faster long-time CO_2_ orientational diffusion in [BMIM^+^]­[PF_6_^–^] while resulting in slower diffusion in [BMIM^+^]­[Tf_2_N^–^]. To quantify this behavior, the OCFs were fit to the sum of a Gaussian-shaped decay function for inertial motions and a stretched exponential decay function for longer times.

2 $$C_{\theta }\left(t\right)={ae}^{-{\left(t/\tau_{1}\right)}^{2}}+\left(1-a\right)e^{-{\left(t/\tau_{2}\right)}^{\beta }}$$

We held β = 0.4 to facilitate direct comparison of the long-time scale behavior. The obtained fitting constants are summarized in Table 4. The short time scale, τ_1_, shows faster orientational decorrelation for the polarizable force field than for the nonpolarizable force field for both ILs. The longer time scale, τ_2_, reveals a more complex pattern of behavior. We observe the fastest orientational diffusion for the nonpolarizable [BMIM^+^]­[Tf_2_N^–^] system and the slowest orientational diffusion for the polarizable [BMIM^+^]­[PF_6_^–^] system. This slowest value should be viewed as a minimum estimate, since there is still substantial orientational correlation at the end of our 1 ns window of observation. The trends in the fitting constants are in agreement with the visual inspection of Figure 5. Given that the caging time is about 4 ps and the values of τ_1_ are less than this, these shorter time scales can be interpreted as the time scale for orientational “rattling” within an ion cage. The larger *a* values for the polarizable force field show that this model allows greater orientational freedom within an ion cage than the nonpolarizable model (Figure 6).

**Table 4:** Orientational Relaxation Time Constants of CO_2_ in [BMIM^+^]­[Tf_2_N^–^] and [BMIM^+^]­[PF_6_^–^]­^a^

	[BMIM^+^][Tf_2_N^–^]		[BMIM^+^][PF_6_^–^]	
system	pol	nonpol	pol^b^	nonpol
*a*	0.647 ± 0.002	0.397 ± 0.003	0.622 ± 0.001	0.469 ± 0.003
τ_1_ (ps)	0.264 ± 0.002	0.302 ± 0.005	0.289 ± 0.003	0.341 ± 0.006
τ_2_ (ps)	21.7 ± 0.4	46.9 ± 0.6	220 ± 2	73.1 ± 1.2
exp. τ_2_^′^ (ps)	31 ± 5		46 ± 9	

a The rotational time constants τ_θ_ are determined by analytically integrating the fitted bi-exponential function.

b The [BMIM^+^]­[PF_6_^–^] system with polarizable force fields does not fully decay in the collected data.

**Figure 6:**
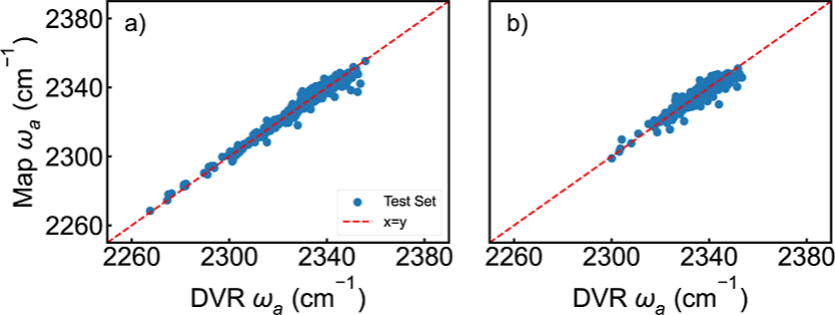
Correlation between the predicted asymmetric vibrational frequencies of CO_2_ from the spectroscopic map with the instantaneous angle frequency effect and the frequencies calculated through DVR calculations for (a) the polarizable force field (*R* = 0.99, RMSD = 2.32 cm^–1^) and (b) the nonpolarizable force field (*R* = 0.94 and RMSD = 2.67 cm^–1^). The red curve indicates a perfect correlation.

The longer τ_2_ constants are all substantially greater than 4 ps, indicating that these constants describe the time taken to achieve complete orientational diffusion after the CO_2_ escapes a specific ion cage. From the aCDFs, we have already found that the effect of explicit polarizability is to soften the CO_2_-anion interactions. One might expect this to lead to the CO_2_ escaping from local cages more quickly, but this is the opposite of the trend observed in the τ_2_ values in the case of [BMIM^+^]­[PF_6_^–^]. It is useful to make comparisons with the OCFs for the IL ions given in the Supporting Information. At short times, the OCFs are identical between polarizable and nonpolarizable force fields. At longer times, the cations show the same pattern of orientational diffusion as is observed for CO_2_, where [BMIM^+^] in nonpolarizable [BMIM^+^]­[Tf_2_N^–^] shows the fastest orientational diffusion and [BMIM^+^] in polarizable [BMIM^+^]­[PF_6_^–^] shows the slowest orientational diffusion. Unexpectedly, the orientational diffusion of the anions does not share this relationship. This may be due to the fact that the anions are generally smaller than the cations and so can rotate without requiring the entire solvent cage to break.

The long time constants, τ_2_, obtained in our simulations can also be compared to long-time anisotropy decay times from experimental data, given in Table 4. In [BMIM^+^]­[PF_6_^–^], the value obtained from the polarizable model (which should be treated as a lower limit) is much larger than the experimental value. Otherwise, the experimental long time scales, τ_2_^′^, are somewhat close to the simulated τ_2_ values. For this observation, explicit polarizability does not improve the model. In the case of [BMIM^+^]­[PF_6_^–^], including polarizability makes the model substantially worse, while for [BMIM^+^]­[Tf_2_N^–^], polarizability nearly reproduces the inaccuracy of the nonpolarizable model but as a negative rather than positive deviation.

### Computational Vibrational Spectroscopy

From each of our four simulated systems, we extracted 1000 snapshots with a time separation of 100 ps for a total of 4000 snapshots using discrete variable representation (DVR). Previously, we developed a spectroscopic map for the asymmetric stretch of a nonpolarizable model of CO_2_ in [BMIM^+^]­[PF_6_^–^].^40^ In this map, we separated the effects of cations from anions and included features related to the electric field and the Lennard–Jones potential, which account for short-range repulsion and dispersion. To compute IR spectra and frequency–frequency correlation functions (FFCFs) for the other three systems in this work, we needed new spectroscopic maps for the polarizable models and for [BMIM^+^]­[Tf_2_N^–^]. To develop a map for the nonpolarizable case, we assumed that we could use the same map features for both ILs. Then, we mixed our 2000 snapshots for this case and randomly removed 500 to act as a test set. With the same features as in our prior work, we used multilinear regression as implemented in scikit-learn to train the spectroscopic map on the 1500 training set snapshot frequencies.^40,41^ For the polarizable force field, we used each type of energy component included in the force field (eq 4), separated into anion and cation contributions and CO_2_ carbon and CO_2_ oxygen contributions as map features. This approach follows a similar design philosophy to that used for the nonpolarizable force field, but adapted to the different structure of the polarizable force field. Again, a randomly selected training set with 1500 members was used along with multilinear regression from scikit-learn to produce the spectroscopic map. Both spectroscopic maps show good performance against their 500-member test sets, and both ILs are accurately described. More details on the spectroscopic maps, including the inclusion of the CO_2_ bend angle, are given in the Methods section and the Supporting Information.

Using these spectroscopic maps, we computed the CO_2_ asymmetric stretch IR spectra for each model using the fluctuating frequency approximation (eq 23).^42^ These predicted IR spectra are shown in Figure 7 alongside the experimental spectra.^14,31^ In Figure 7a,b, the instantaneous angle contribution, Δω_θ_(*t*), was removed from each frequency before computing the IR spectrum, and then the final spectra were shifted by the average angle contribution ⟨Δω_θ_⟩ = 2.7 cm^–1^ found in our prior work.^40^ The instantaneous angle contribution was included in the calculation of the spectra in Figure 7c,d. The peak frequencies and full width at half-maxima (fwhm) are summarized in Table 5.

**Figure 7:**
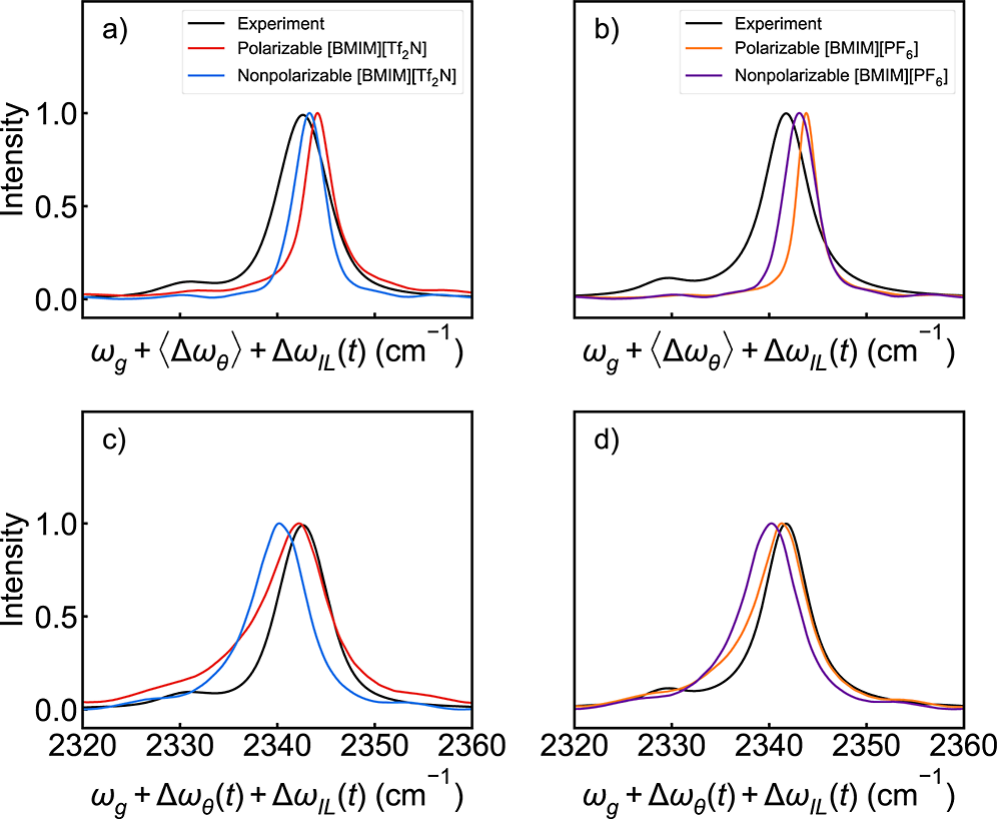
IR spectra for the asymmetric stretch of a CO_2_ molecule solvated in polarizable and nonpolarizable models of (a) [BMIM^+^]­[Tf_2_N^–^] and (b) [BMIM^+^]­[PF_6_^–^] calculated using the average angle contribution, ⟨Δω_θ_⟩, and (c) [BMIM^+^]­[Tf_2_N^–^] and (d) [BMIM^+^]­[PF_6_^–^] calculated using the instantaneous angle contribution, Δω_θ_(*t*). Experimental data are included for comparison.^14,31^

**Table 5:** Peak Frequencies and FWHMs for Experimental and Computational Spectra^a^

system	[BMIM^+^][Tf_2_N^–^]			[BMIM^+^][PF_6_^–^]		
	exp	pol	nonpol	exp	pol	nonpol
peak, ⟨Δω_θ_⟩	2342.64	2344.13	2343.30	2341.74	2343.80	2343.09
fwhm, ⟨Δω_θ_⟩	6.15	3.47	3.75	5.48	2.46	3.90
peak, Δω_θ_(*t*)	2342.64	2342.24	2340.19	2341.74	2341.29	2340.23
fwhm, Δω_θ_(*t*)	6.15	7.94	6.57	5.48	6.27	6.65

a Simulated spectra are computed with the average contribution from the OCO angle, ⟨Δω_θ_⟩, or with the instantaneous contribution from the OCO angle, Δω_θ_(*t*). All values are given in wavenumbers, cm^–1^.

All computational spectra using the average angle ⟨ω_θ_⟩ are narrower than that in the experiment. In Figure 7a,b, the nonpolarizable and polarizable models have very similar predicted spectra, but the nonpolarizable cases produce a peak slightly closer to the main experimental peak. The spectra from nonpolarizable models are peaked at slightly lower frequencies than the spectra from polarizable models, both of which have peaks which are blue shifted with respect to the experiment. The polarizable models reliably produce narrower fwhm values than the nonpolarizable models. Overall, and surprisingly, the nonpolarizable models agree slightly better with the experiment than the polarizable models.

The situation is somewhat different when the instantaneous angle Δω_θ_(*t*) is included in the calculation. The peaks are all slightly to the blue of the experiment, but the polarizable model predictions are very close to those of the experiment. The experimental fwhm is smaller than any of the computational predictions, but always by less than 2 cm^–1^. Overall, when the instantaneous angle is included, the simulated IR spectra are more accurate in the polarizable case. None of the models directly predict the second peak near 2330 cm^–1^ shown in the experimental spectra. This is expected since the spectroscopic maps do not contain any model for the hot band that produces this peak in the experiment.^31^ However, when the instantaneous angle is included as in Figure 7c,d, the peaks in the IR spectra skew toward the red. This is because the CO_2_ angle is treated classically in our simulations. This effect should produce overestimates of the breadth of the main IR spectra compared to experiments. This is seen in our data.

The linear IR spectrum is strongly related to the structure of CO_2_ in the IL but weakly related to its solvation dynamics. Experimentally, additional information on the solvation dynamics can be extracted with 2D-IR experiments.^31^ Using analysis techniques such as the center line slope, the frequency–frequency correlation function (FFCF) given by eq 21 can be estimated. This function tracks the randomization of the CO_2_ asymmetric stretch frequency over time.^43,44^ Since the frequency is directly related to the local structure, the FFCF sensitively tracks the dynamics of the local solvent. The CO_2_ asymmetric stretch can be used as a vibrational probe, meaning that experiments can use the resulting 2D-IR spectrum as a detailed reporter of the solvation dynamics of CO_2_.^31^ Computationally, the FFCF can be computed directly from the CO_2_ asymmetric stretch frequency trajectory used to compute the linear IR spectrum (eq 21).^14^ These results are shown in Figure 8.

**Figure 8:**
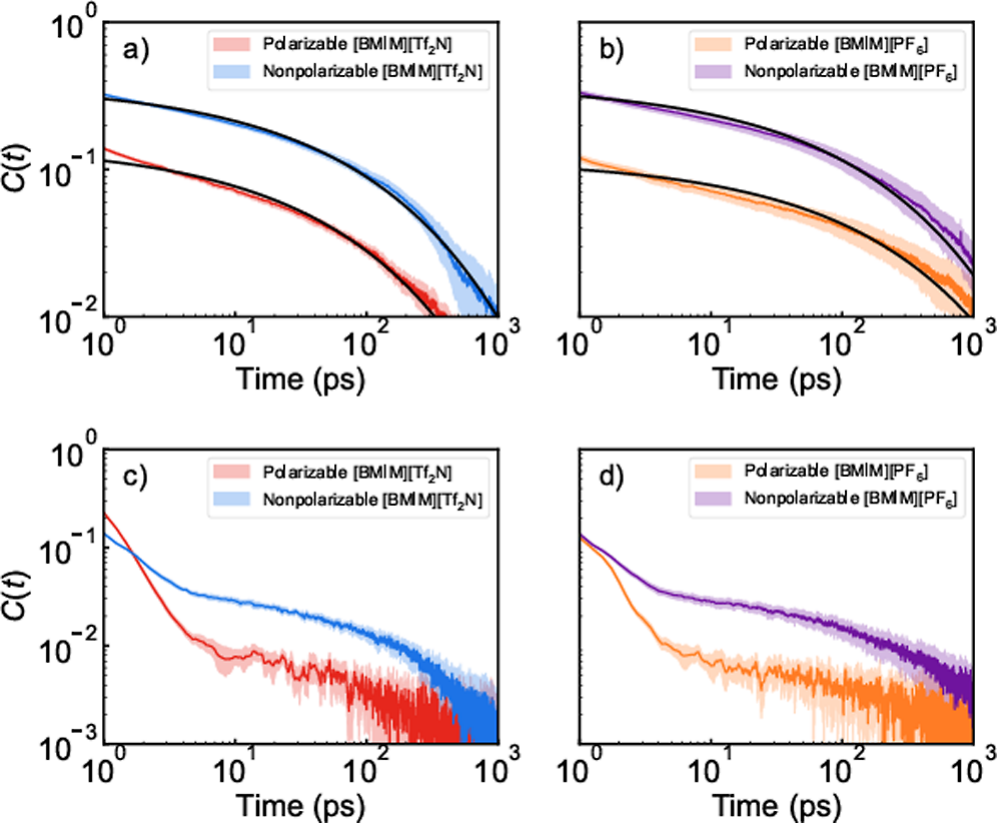
Frequency–frequency correlation function for a CO_2_ molecule solvated in polarizable and nonpolarizable models of (a) [BMIM^+^]­[Tf_2_N^–^] and (b) [BMIM^+^]­[PF_6_^–^] calculated using the average angle contribution, ⟨Δω_θ_⟩, and (c) [BMIM^+^]­[Tf_2_N^–^] and (d) [BMIM^+^]­[PF_6_^–^] calculated using the instantaneous angle contribution, Δω_θ_(*t*). The shaded region represents the 95% confidence interval of the collected data. Fitted curves are shown in black.

We calculated FFCFs for both the average angle (Figure 8a,b) and instantaneous angle (Figure 8c,d) conditions. For the average angle condition, the polarizable models show faster initial decay rates. This is similar to what is observed for the crystalline OCFs. For both ILs, the difference in spectral decorrelation between polarizability conditions decreases at longer times, but the FFCFs do not intersect within 1 ns. The average angle FFCFs were fit using the same function as the OCFs (eq 2), again holding β = 0.4 to ensure the time constants are comparable. The resulting fitting constants are listed in Table 6.

**Table 6:** Spectral Diffusion Time Constants of CO_2_ in [BMIM^+^]­[Tf_2_N^–^] and [BMIM^+^]­[PF_6_^–^] Obtained from Frequency–Frequency Correlation Function^a^

system	[BMIM^+^][Tf_2_N^–^]		[BMIM^+^][PF_6_^–^]	
	pol	nonpol	pol	nonpol
*a*	0.855 ± 0.001	0.627 ± 0.002	0.900 ± 0.001	0.666 ± 0.001
τ_1_ (ps)	0.179 ± 0.001	0.191 ± 0.002	0.176 ± 0.001	0.216 ± 0.001
τ_2_ (ps)	29.4 ± 0.6	40.9 ± 0.5	145 ± 3	88.3 ± 0.8
exp. τ_2_^′^ (ps)	15 ± 1		93 ± 3	

a The FFCF time constants τ_ω_ are determined by analytically integrating the fitted function.

As observed in Figure 8, the amplitude of the initial decay captured by *a* is substantially larger in explicitly polarizable solvents. The τ_1_ decay is less than 4 ps, indicating that this decay results from rattling within the local cage. Like in the OCFs, the short-time decay makes up the majority of the overall decay, but for the FFCFs, this is especially pronounced. Unlike the case for the OCFs, the majority of the nonpolarizable decay also originates from the short-time decay, which speaks to the degree to which the vibrational frequency is sensitive to the local cage. These time scales tend to be shorter than the equivalent OCF time scale. This is because motions of the ions in a given local cage can change the frequency without any CO_2_ rotation through librational “wobbling” motions, but any CO_2_ rotation within a cage will change the frequency. So, within a local cage, the frequency must randomize faster than the orientation.

The pattern in the longer τ_2_ decay times is similar to that observed for the τ_2_ decay times observed for the OCFs. As observed elsewhere in this work, the long-term decay is especially slow for polarizable [BMIM^+^]­[PF_6_^–^]. Besides this case, the FFCF τ_2_ decay times are generally similar to those from the OCFs. Again, this likely describes the gating of the CO_2_ by the overall reorganization of the IL. The comparison between OCF and FFCF τ_2_ values indicates that the CO_2_ orientation randomizes as the local cage is broken, since there is a strong relationship between the FFCF and solvent cage dynamics.^13,30^

The instantaneous angle FFCFs clearly show nonexponential decay behavior. The decay prior to 10 ps is very similar to the OCO angle correlations shown in Figure S5 in the Supporting Information. After this, the FFCFs are broadly parallel to their average angle counterparts in our log–log plots, meaning that they exhibit the same decay time scales. This implies that the CO_2_ angle fluctuations decorrelate the frequency but otherwise the motions responsible for the FFCF decay in the average angle case decorrelate the frequency in the instant angle case.

Comparison to experiment for these data is difficult due to the effects of motional narrowing, which cannot be fully resolved in the experiment. As seen in prior work, our FFCF fitting parameters cannot be compared directly to CLS data except under specific conditions, which are not satisfied by CO_2_ in ILs.^14,43,44^ Even so, we can make some tentative comparisons. For instance, we previously found that the shortest CLS time scale predicted by a nonpolarizable model for CO_2_ in [BMIM^+^]­[PF_6_^–^] was significantly longer than the experimental value.^14^ In this work, we find that explicit polarizability causes faster spectral diffusion at short times, indicating that the polarizable force field shows better agreement with the experiment. Focusing on the longer τ_2_ time scales, which are more directly accessible from the experiment, we see the best agreement for polarizable [BMIM^+^]­[Tf_2_N^–^] and nonpolarizable [BMIM^+^]­[PF_6_^–^]. Additionally, the long-time decay was experimentally found to be much slower for [BMIM^+^]­[PF_6_^–^] than for [BMIM^+^]­[Tf_2_N^–^]. In the simulations, we only observe a slowdown similar to this for the polarizable force field, implying that the polarizable force field is more accurate. In future work, we plan to compute 2D-IR spectra in order to facilitate an apples-to-apples comparison.

## Discussion

### Structure

In our structural analyses, the ion cages surrounding CO_2_ consistently soften when the polarizability is modeled explicitly. In the aCDFs, the polarizable models show smaller anion peaks along with a steep decrease in the cation ring density within the local CO_2_ solvation environment. Ion counting likewise shows that fewer charged moieties are in the vicinity of the CO_2_. The MSD exponent analysis also reveals that the size of the local ion cages is larger in the polarizable model. One might speculate that this could be attributed to the bulk IL density. If the polarizable force fields consistently led to lower neat IL densities, there might be more empty molar volume for the CO_2_ to occupy. However, the neat IL densities predicted by the polarizable models (which show better agreement with experiment) are not consistently smaller than the densities from the nonpolarizable models, as shown in Table 7. This means that the differences in solvation structure between the two models are more likely due to differences in the way that the CO_2_ interacts with the IL ions.

**Table 7:** Density of CO_2_-In-IL Systems Studied in This Work^a^

system	[BMIM^+^][Tf_2_N^–^]			[BMIM^+^][PF_6_^–^]		
	exp	pol	nonpol	exp	pol	nonpol
density (g cm^–1^)	1.438	1.406 ± 0.005	1.490 ± 0.003	1.370	1.388 ± 0.004	1.297 ± 0.004

a Experimental values are taken from the literature.^32,45,46^ The presence of a single CO_2_ molecule does not appreciably change the overall density.

The CO_2_ forms its local ion cages through two mechanisms: enthalpically via its molecular quadrupole and entropically by occupying pre-existing voids in the IL.^13,20^ Explicit polarizability weakens the enthalpic effect by reducing the effective instantaneous quadrupole of the CO_2_ molecule. As a result, the surrounding ion cages become larger and the charged portions of cations are less present. However, Drude particles offer only an intermediate treatment of polarization. Ab initio molecular dynamics (AIMD) simulations of imidazolium-based ILs provide a generally better treatment of polarization and charge transfer than either force field used here. However, their steep cost requires short simulation times. Even so, they can be useful for investigating molecular structure and provide a useful benchmark here. Prior simulations of this kind show strong interactions between the CO_2_ carbon and the anion, as is observed here.^47–49^ Important secondary interactions between CO_2_ and the cation are also observed. However, these are more varied—the CO_2_ often interacts dispersively with the less charged portions of the cation or with the π system of the imidazolium ring.^47,49^ Interactions between the CO_2_ oxygen and the cation ring are still important, but they are not the only or even main kind of CO_2_–cation interaction.^47^ In this work, the polarizable force field better captures this variety of cation interactions.

### Dynamics

Dynamical analyses included t-qCDF, MSD, and OCF. In the nonpolarizable models, the charge density of the ions near the CO_2_ does not begin to change substantially until after a roughly 100 ps waiting period in both [BMIM^+^]­[Tf_2_N^–^] and [BMIM^+^]­[PF_6_^–^]. In the polarizable [BMIM^+^]­[Tf_2_N^–^] case, a small amount of residual ion density was found after 100 ps, while in the polarizable [BMIM^+^]­[PF_6_^–^], the anion–CO_2_ interaction lasts beyond 1000 ps. The small value of the residual ion density for [BMIM^+^]­[Tf_2_N^–^] at 100 ps is mainly an artifact of the reduced ion density at 0 ps. Relative to 10 ps, the charge intensity at 100 ps for the polarizable [BMIM^+^]­[Tf_2_N^–^] model had decayed by a similar amount to that in the nonpolarizable [BMIM^+^]­[Tf_2_N^–^] model.

The results of the OCF offer additional insight into the CO_2_ dynamics in ILs. At short times, the CO_2_ molecule shows a slightly faster dynamics in the polarizable models. At long times, however, the dynamics differ substantially among the four cases studied in this work. In our prior work, it was shown that the orientational motions of the CO_2_ in an IL are gated by the local ion cage.^13,30^ Our current results are consistent with this picture for both force field types. The faster short time scale in the polarizable model is explained by the local IL cage structure. Looser cages in the polarizable case offer more opportunities for localized wobbling before the presence of other ions stops rotational motion. This is also consistent with the longer caging times found in the polarizable models from our MSD results.

The slower orientational relaxation time constant and slower diffusion constant in the polarizable model can be traced back to changes in the overall dynamics of ILs induced by explicit polarizability. While explicit polarizability often increases the speed of the large-scale IL reorganization for most ILs,^27,33,50,51^ the opposite trend has been observed in [BMIM^+^]­[PF_6_^–^] where explicit polarization actually slows ion diffusion down.^32,33,52,53^ Therefore, the long-term dynamic behavior of the CO_2_ in the ILs is most related to the structural reorganization of the IL. The overall picture is one where the CO_2_ rattles in a local cage until the entire liquid reorganizes, trapping it in a new cage.

### Spectroscopy

In our previous work, we found that lower CO_2_ vibrational frequencies were associated with tighter ion cages.^14^ Since the polarizable models predict larger ion cages, it makes sense that they also produce blue-shifted IR spectra. When the instantaneous angle is included in their calculation, these IR spectra based on polarizable models are in stronger agreement with the experiment than those predicted by the nonpolarizable model. However, both cases are generally similar to each other and to the experiment, differing at most in their peak frequencies by ∼2 cm^–1^. Explicit polarizability does not correct the too narrow IR spectrum predicted by the nonpolarizable models. Instead, including the instantaneous angle is sufficient to capture the line width for both models and both ILs. Spectral diffusion is also altered by the inclusion of explicit polarizability. As observed for the OCF, short-time spectral diffusion is sped up, while long-time spectral diffusion appears to be more system-dependent. We look forward to computing the 2D-IR spectra needed to fully validate these comparisons.

## Conclusion

In this work, we employed explicitly and implicitly polarizable models of the solvation of CO_2_ in ILs. Using these models, we computed structural, dynamic, and spectroscopic properties describing the solvation of CO_2_. We found that the local ion cage structure around the CO_2_ was substantially changed by explicit polarization, as were the dynamical properties. Where experimental values for the CO_2_ structural, dynamical, and spectroscopic properties were available, greater agreement with experiment is usually, but not always, observed for the explicitly polarizable model. In most of these cases, the improvement over the nonpolarizable model is modest. However, direct comparisons between experiment and theory are limited for the FFCFs because of motional narrowing, which cannot be resolved in the experiment.

Altogether, we believe that simulations without explicit polarizability can be used to cheaply compute CO_2_-in-IL IR spectra with relatively little loss in accuracy. Even so, the structural and dynamical properties predicted by these force fields should be viewed with some suspicion. One should adjust interpretations to include more varied CO_2_-cation interactions, faster short-time inertial motion, and long-time diffusion more like the bulk IL than a nonpolarizable charge-scale model would predict.

## Methods

### Force Fields

#### Nonpolarizable Force Field

The parameters for the nonpolarizable force field were adapted from the Optimized Potentials for Liquid Simulations—All Atom (OPLS-AA) force field for the ions ([BMIM^+^], [Tf_2_N^–^], and [PF_6_^–^]).^54,55^ To implicitly account for electronic polarization effects, all ion charges were scaled by 0.8. The transferable potentials for phase equilibria model was used for the CO_2_ molecule.^56^ Bonded parameters for a flexible CO_2_ were borrowed from Perez–Blanco and Maginn.^57^ In this force field, the potential energy function is expressed as

3 $$E=\sum_{bonds}\frac{k_{x}}{2}{\left(x-x_{0}\right)}^{2}+\sum_{angles}\frac{k_{\theta }}{2}{\left(\theta -\theta_{0}\right)}^{2}+\sum_{dihedrals}k_{\phi }\left(1+\cos\left(n\phi -\phi_{0}\right)\right)+\sum_{i}^{N-1}\sum_{j=i+1}^{N}\left[4\varepsilon_{ij}\left({\left(\frac{\sigma_{ij}}{r_{ij}}\right)}^{12}-{\left(\frac{\sigma_{ij}}{r_{ij}}\right)}^{6}\right)+\frac{q_{i}q_{j}}{4\pi \epsilon_{0}r_{ij}}\right]$$

The first three terms describe bond, angle, and dihedral intramolecular interactions. The first two sums in eq 3 describe simple harmonic oscillator models where *k*_*x*_ and *x*_0_ represent the force constant and the equilibrium bond length, respectively, and *k*_θ_ and θ_0_ represent the force constant and the equilibrium angle of an angular interaction, respectively. The third term in eq 3 represents the periodic torsion force associated with the rotational motion around bonds where *k*_ϕ_ is the torsion force constant, *n* is the periodicity, ϕ is the dihedral angle, and ϕ_0_ is the phase offset.

The final term describes interactions between atoms that are not bonded to each other or are more than four bonds away from each other. These include short-range repulsions and dispersive attractions between atoms using the Lennard–Jones potential and electrostatic interactions using the Coulomb potential. Atoms separated by four bonds also experience these interactions but reduced in magnitude by a factor of 2. The indices *i* and *j* here represent two different atoms, σ_*ij*_ denotes the cross-interaction diameter, ε_*ij*_ is the energy interaction parameter, *r*_*ij*_ is the distance between the two atoms, *q*_*i*_ and *q*_*j*_ are the partial charges for atom *i* and atom *j*, respectively, and ϵ_0_ is the permittivity of free space. The cross-interaction diameter and the energy interaction parameter are estimated using the Lorentz–Berthelot mixing rules on single atom parameters, i.e., $\sigma_{ij}=\frac{\sigma_{ii}+\sigma_{jj}}{2}$ and $\epsilon_{ij}=\sqrt{\epsilon_{ii}\epsilon_{jj}}$ .

#### Polarizable Force Field

The parameters for the polarizable force field were taken from the Symmetry-Adapted Perturbation Theory (SAPT)-based force field for ILs developed by McDaniel et al. and for CO_2_ developed by Yu et al.^32,58^ Bonding parameters for a flexible CO_2_ were again borrowed from Perez-Blanco and Maginn.^57^ In this force field, the bonded terms in the potential energy function (bonds, angles, and dihedrals) are defined using largely conventional functional forms, as in eq 3.

The nonbonded energy is a composition of energy terms given as follows

4 $$E_{non‐bonded}=E_{pol}+E_{elec}+E_{exch}+E_{ind}+E_{disp}+E_{\delta hf}$$

where the terms describe polarization (*E*_pol_), electrostatic interactions (*E*_elec_), exchange repulsion (*E*_exch_), induction (*E*_ind_), dispersion (*E*_disp_), and higher-order contributions to polarization and/or exchange that are not captured in the previous terms (*E*_δhf_). The complete details for modeling these interactions are provided in the work of McDaniels, Schmidt, Yethiraj, and their co-workers.^32,34,58–60^ For some of our spectroscopic mappings, we calculated these terms for individual atomic interactions and used them as features.

#### Molecular Dynamics Simulations

Molecular dynamics simulations were performed using OpenMM 8.1.1 with CUDA 11.8.0 on single NVIDIA RTX 6000 GPUs.^61^ Simulations were run using polarizable and nonpolarizable force fields for CO_2_ in the IL solvents [BMIM^+^]­[Tf_2_N^–^] and [BMIM^+^]­[PF_6_^–^], for four simulated systems in total.

For all simulations, initial atomic coordinates were generated using the PACKMOL software.^62^ A single CO_2_ molecule was placed near the center, and 256 ionic liquid pairs were randomly positioned in a cubic simulation box with edge lengths of 45 Å. The edge lengths of the simulation box were manually increased to 46 Å after packing without moving any atoms, to avoid harmful contacts due to periodic boundary conditions. The energy of the structure was then minimized by using the desired force field. Long-range electrostatic interactions beyond 15 Å were approximated by using the particle mesh Ewald method. After energy minimization, the system was equilibrated at constant volume and constant temperature for 1 ns using a Langevin thermostat with a coupling constant of 1 ps^–1^ at 300 K with a time step of 1 fs. For the polarizable systems, the Drude particles were simulated using a dual Langevin integrator with a coupling constant of 1 ps^–1^ at 1 K. The maximum distance between Drude particles and their heavy atoms was set to 0.2 Å to prevent a polarization catastrophe.^63^ After the initial equilibration simulation, the system was equilibrated for 3 ns at a constant pressure and constant temperature. The pressure was held at 1 bar by a Monte Carlo barostat with a barostat frequency of 0.1 ps^–1^. Following this, the system was heated from 300 to 600 K over 1 ns, then cooled back to 300 K over 1 ns, all at constant volume. The heating step breaks up long-lived ion cages in the liquid. Finally, the system equilibrated at 300 K for another 1 ns under a constant volume and constant temperature.

For each system, ten production simulations were run using a Langevin thermostat at a constant temperature of 300 K for 100 ns. For CDF, OCF, DVR, and IR analysis, configurations were saved at 0.25 ps intervals to ensure sufficient temporal resolution for capturing the structural and dynamical behaviors of CO_2_ in ILs. For MSD calculations, five CO_2_ molecules were placed within the box to ensure sufficient sampling of diffusion statistics. These CO_2_ molecules were initially placed randomly in the simulation box, and the system was subjected to the same equilibration and production protocols described above. The resulting 100 ns production simulation was saved at 1 ps intervals.

### Data Analysis

#### Cylindrical Distribution Function

The cylindrical distribution function (CDF) measures the particle density in two dimensions around a central location with cylindrical symmetry.^13^ Since the CO_2_ molecule is cylindrically symmetric, this analysis method reveals aspects of its solvation that are not clear in the more traditional radial distribution function.^25^ In the CDFs used here, the space of interest is divided into cylindrical bins organized by their height above the CO_2_ carbon and their radius in the direction orthogonal to the CO bond. The radius, *r*, and the height, *z*, of each cylindrical bin can be calculated as follows

5 $$z={\mathrm{v̂}}_{CO}\cdot {\mathrm{v⃗}}_{Ci}$$

6 $$r=\left|{\mathrm{v⃗}}_{Ci}-z{\mathrm{v̂}}_{CO}\right|$$

where ${\mathrm{v̂}}_{CO}$ is the unit vector in the direction of a CO bond and ${\mathrm{v⃗}}_{Ci}$ is the vector connecting the carbon atom of the CO_2_ molecule to the atom of interest. To increase our sampling, both CO bond vectors were used to calculate individual CDFs, and then the results were averaged together.

We perform two types of CDF analyses to analyze the distribution of atoms and charges. The first is the atomistic cylindrical distribution function (aCDF). In this version, a specific atom type is selected and counted in each CDF bin. Alternatively, the charges of all atoms are binned in the charge-cycle distribution function (qCDF). Both can provide information on the solute–solvent interactions between the CO_2_ molecule and the IL. In both CDF types, the cylindrical shell is divided into small bins as described above. The aCDF at a particular height, *z*, and radius, *r*, is given by

7 $$g\left(r,z\right)=\frac{\left\langle \rho \left(r,z\right)\right\rangle }{\rho }$$

where ⟨ρ­(*r*, *z*)⟩ is the average number density of the particle of interest in the cylindrical bin ranging from (*r*, *z*) to (*r* + Δ*r*, *z* + Δ*z*), averaged across all the collected snapshots and ρ is the bulk density of the particle of interest. To simplify our calculations, we always chose Δ*r* = Δ*z*. The term ρ­(*r*, *z*) can be expressed as

8 $$\rho \left(r,z\right)=\frac{\left\langle N\left(r,z\right)\right\rangle }{V_{bin}\left(r,z\right)}$$

where ⟨*N*(*r*, *z*)⟩ is the average number of particles of interest in the bin at (*r*, *z*) and *V*_bin_(*r*, *z*) is the volume of that bin, which is

9 $$V_{bin}\left(r,z\right)=V\left(r+\Delta r,z+\Delta z\right)-V\left(r,z\right)=\pi \left(2r\Delta r^{2}+\Delta r^{3}\right)$$

where the volume formula for a cylinder is used to calculate *V*(*r*, *z*). For the qCDF, charges were binned instead of particle numbers

10 $$g\left(r,z\right)=\frac{\left\langle q\left(r,z\right)\right\rangle }{V_{bin}\left(r,z\right)}$$

where *q*(*r*, *z*) is the sum of charges in the cylindrical bin ranging from (*r*, *z*) to (*r* + Δ*r*, *z* + Δ*z*). In many cases, we were interested in the dynamic evolution of the solvent cage around the CO_2_. To examine this, we calculated time-dependent charge cylindrical distribution functions. Atomic charges were binned only if they were within the CO_2_ solvation cage (|*z*| ≤ 5 Å and *r* ≤ 5 Å) at time *t* and *t* + τ, where τ is a time separation.

#### Orientation Correlation Function

The orientation correlation function (OCF) describes the rotational motion of a molecule. It can be obtained using the relationship^29^

11 $$C_{\theta }\left(\tau \right)={\left\langle P_{2}\left[\mathrm{r̂}\left(t+\tau \right)\cdot \mathrm{r̂}\left(t\right)\right]\right\rangle }_{t}$$

where *P*_2_ is the second Legendre polynomial, *r̂*(*t*) is a molecular orientation unit vector, τ is a time separation, and *t* is the initial time. The correlation for a specific time separation τ is found by averaging over all possible initial times, *t*. At short time separations, the initial direction and final direction are highly correlated; therefore, *C*_θ_(0) = 1. At long time separations, the final orientation is unrelated to the original orientation; therefore, *C*_θ_(∞) → 0. This can be used as a key indicator to characterize molecular dynamics within a liquid environment. If the orientation becomes more randomly distributed, then *C*_θ_ will approach the value of 0. This orientational correlation function can also be integrated to provide the average time required for a molecule to lose its memory of its initial orientation.

12 $$\tau_{\theta }=\int_{0}^{\infty }C_{\theta }\left(\tau \right)d\tau$$

#### Self-Diffusivity

The diffusion coefficient, describing the self-diffusivity of each molecule in the simulation, was calculated based on the mean squared displacement (MSD)

13 $$MSD\left(\tau \right)={\left\langle |\mathrm{r⃗}\left(t+\tau \right)-\mathrm{r⃗}\left(t\right){|}^{2}\right\rangle }_{t}$$

where τ is a time separation, *r⃗*(*t*) is the center of mass vector for the molecule of interest, and the average is taken over all available starting times, *t*. The diffusion coefficient is determined from the slope of the MSD as a function of the time separation, τ, once the MSD reaches the linear diffusive regime at large time separations.

14 $$D=\frac{1}{6}\lim_{\tau \to \infty }\frac{d}{d\tau }MSD\left(\tau \right)$$

In this work, we evaluate the slope between τ = 0.4 ns and τ = 4.0 ns since all molecules investigated are considered to be in the diffusive regime by that point.

### IR Spectroscopic Analysis

#### Discrete Variable Representation Calculation

Snapshots from the simulation trajectories were analyzed using a discrete variable representation (DVR) approach applied to the CO bond stretching potential energy surface (PES).^64–66^ The PES along the bond lengths of CO_2_ was determined using single-point energy calculations with *Q*-Chem 5.4.^64,67^ One thousand independent snapshots were extracted from the production simulations run for each system to obtain CO_2_-IL clusters, including two ion pairs represented with density functional theory and the surrounding ionic liquid environment represented with molecular mechanics region in the same manner used by Daly et al.^40^ For each extracted snapshot, the CO_2_ geometry was discretized into a 10 × 10 matrix (i.e., 100 grid points) by varying each CO bond length between 0.98 and 1.35 Å with a step size of 0.041 Å. This range of CO bond lengths was chosen by testing an array of possible ranges on a single CO_2_-[BMIM^+^]­[PF_6_^–^] cluster as shown in the Supporting Information. The resulting anharmonic vibrational frequency was found to be invariant near the chosen range. Molecular energy calculations were then performed using B3LYP/6-311++G** until the self-consistent field error converged to 10^–10^ a.u. to construct the Born–Oppenheimer PES for the CO_2_ molecule in each snapshot.^68,69^

The resulting DVR frequencies were scaled by 0.985165 to account for the effects of basis set incompleteness and inaccuracies in the employed density functionals. This scaling factor was derived from the ratio between the predicted asymmetric stretch frequency of gas-phase CO_2_ and the experimental gas-phase CO_2_ asymmetric stretch frequency.

#### Spectroscopic Mapping

We consider the asymmetric stretch frequency to be a combination of the gas-phase asymmetric stretch frequency and frequency-shifting factors resulting from both the OCO angle and the IL solvent, given by

15 $$\omega_{a}=\omega_{g}+\Delta \omega_{\theta }+\Delta \omega_{IL}$$

where ω_*a*_ is the calculated asymmetric stretch frequency of a CO_2_ solvated in an IL, ω_*g*_ is the experimental asymmetric stretch frequency for gas-phase CO_2_ (2349.1 cm^–1^), Δω_θ_ is the frequency change caused by changes in the OCO angle, and Δω_IL_ is the frequency change caused by the presence of the IL solvent.^40,70,71^

In prior work, we held the CO_2_ rigid during simulations using a setting in the LAMMPS program.^72^ To improve our sampling, we currently use the GPU-accelerated OpenMM program.^61^ Unfortunately, OpenMM does not have the capability to fix the bond lengths and angle of the linear CO_2_ while also modeling Drude particles. This necessitates the use of a flexible CO_2_ model in explicitly polarizable simulations. For consistency, we also use a flexible CO_2_ in the simulations based on nonpolarizable models. Because the angle is modeled classically in our simulations, it produces a long tail to the left of the asymmetric stretch frequency distribution.^40^ In the experiment, the angle instead shows up as a hot band.^31^

For this work, we tested two approaches to the inclusion of the angle in the spectroscopic map. First, we assume the angle exists in its vibrational ground state and include only its average effect on the frequency using

16 $$\omega_{a}=\omega_{g}+\left\langle \Delta \omega_{\theta }\right\rangle +\Delta \omega_{IL}\left(t\right)$$

For this approach, we assume that the average angle contribution ⟨Δω_θ_⟩ to be 2.7 cm^–1^ based on prior work.^14,40^ Second, we include the frequency effect from the instantaneous angle in each snapshot (eq 17).

17 $$\omega_{a}=\omega_{g}+\Delta \omega_{\theta }\left(t\right)+\Delta \omega_{IL}\left(t\right)$$

For either approach, we first map the relationship between the angle and asymmetric stretch frequency with the equation

18 $$\Delta \omega_{\theta }\left(t\right)=a\left(1+\cos\left(\theta \left(t\right)\right)\right)$$

where *a* = −1164.7 cm^–1^ is determined by linear regression with no intercept for calculations on a gas-phase CO_2_ at different angles θ.^40^ With this function, we can calculate and isolate the instantaneous Δω_θ_(*t*) effect in every snapshot.

Multilinear models were employed to construct empirical spectroscopic maps for the solvent-induced frequency shift of the asymmetric stretch of CO_2_ in ILs, Δω_IL_. For the nonpolarizable force field, this solvent effect is modeled based on our previous work^40^ using

19 $$\Delta \omega_{IL,nonpol}=b_{1}E_{O}^{cation}+b_{2}E_{O}^{anion}+c_{1}U_{O}+c_{2}U_{C}$$

Here, *E*_O_^cation^ and *E*_O_^anion^ are the electric fields due to the surrounding cations and anions, respectively, across the CO_2_ bonds. *U*_O_ and *U*_C_ represent the Lennard–Jones potential energy contribution at the oxygen and carbon atoms of CO_2_. The values *b*_1_, *b*_2_, *c*_1_, and *c*_2_ are fitting coefficients determined through multilinear regression. For the polarizable force field, the mapping features were also based on the nonbonded interactions. Each energy component was partitioned into contributions from interactions between the surrounding cations or anions and the oxygen or carbon atoms of CO_2_. The complete functional form of the solvent-induced portion of the spectroscopic map for the polarizable force field is given by

20 $$\Delta \omega_{IL,pol}=\sum_{j}^{2}\left(a_{ij}E_{elec}+b_{ij}E_{exch}+c_{ij}E_{disp}+d_{ij}E_{\delta hf}\right)$$

where the index *i* runs over the two types of atoms in a CO_2_ molecule (carbon and oxygen) and *j* runs over the two types of ions in the solvent (cations and anions). The constants *a*_*ij*_, *b*_*ij*_, *c*_*ij*_, and *d*_*ij*_ are fitting coefficients corresponding to the energy components of electrostatic (*E*_elec_), exchange (*E*_exch_), dispersion (*E*_disp_), and higher-order contributions (*E*_δhf_).

#### Frequency–Frequency Correlation Functions

Frequency–frequency correlation functions (FFCFs), which describe how the vibrational frequency fluctuates over time, were computed for both the nonpolarizable and polarizable systems in this work. The correlation function *C*(τ) is obtained using

21 $$C\left(\tau \right)=\frac{\left\langle \delta \omega \left(t\right)\delta \omega \left(t+\tau \right)\right\rangle }{\left\langle \delta \omega \left(t\right)\delta \omega \left(t\right)\right\rangle }$$

where

22 $$\delta \omega \left(t\right)=\omega \left(t\right)-{\left\langle \omega \right\rangle }_{t}$$

Here, ω­(*t*) and ω­(*t* + τ) are the instantaneous asymmetric stretch vibrational frequencies of CO_2_ at time *t* and *t* + τ, respectively, where τ represents a time separation between two frequency observations and ⟨ω⟩_*t*_ represents the time-averaged vibrational frequency over the entire simulation.

#### Linear Infrared Spectra

The IR spectra of CO_2_ were computed using the fluctuating frequency approximation, given by

23 $$I\left(\omega \right)\propto R\left[\int_{0}^{\infty }dte^{i\omega t}\left\langle \mathrm{\mu ⃗}\left(t\right)\cdot \mathrm{\mu ⃗}\left(0\right)e^{-i\int_{0}^{t}d\tau \delta \omega \left(\tau \right)}\right\rangle e^{-t/2T_{1}}\right]$$

where *I*(ω) is the spectral intensity at frequency ω, μ⃗(*t*) is the transition dipole moment vector represented by the normalized CO bond vector per the Condon approximation,^40^ δω­(τ) = ω­(*t*) – ⟨ω⟩_*t*_ is the trajectory of frequency fluctuations, and *T*_1_ is the vibrational population lifetime taken as 58 ps from experimental results.^14,42,73^ In this work, the Fourier-transform infrared spectroscopy (FTIR) spectrum was computed from ten 100 ns production simulations, with snapshots collected every 0.25 ps.

### Experimental Methods

#### Sample Preparation

1-Butyl-3-methylimidazolium bistriflimide 99% and 1-butyl-3-methylimidazolium hexafluorophosphate 99% were obtained from IoLiTec Inc. and used without further purification. Before use, 1 mL of the IL was dried under vacuum at 75 °C for 1 h to remove excess water. Bone dry carbon dioxide (99.8% pure, Matheson Trigas, Inc.) was flowed over the IL for 15 min. One microliter of IL was then placed between two calcium fluoride windows (Crystran Ltd., UK) separated by a 12 μm polytetrafluoroethylene spacer (Harrick Scientific) and housed in a homemade brass cell.^74^

#### Fourier Transform Infrared Spectroscopy

FTIR spectra of dry samples and CO_2_-loaded samples were collected at room temperature with a nitrogen-purged 6700 Nicolet Spectrometer (ThermoFisher Scientific) at a resolution of 0.5 cm^–1^. Samples were prepared such that the ν_3_ mode of CO_2_ was between 0.2 and 0.4 OD.

#### 2-Dimensional Infrared Spectroscopy

A detailed description of the 2D-IR instrument is described elsewhere.^75^ In brief: a commercial titanium/sapphire amplifier system (Coherent Vitesse/Legend Elite) produced 5 kHz of 800 nm, 120 fs pulses that go into a home-built optical parametric amplifier (OPA).^76^ The 800 nm pulses are down-converted into around 1 μJ of 4.5 μm, 200 fs pulses of IR light. This IR light is split into pump and probe pulses. The smaller portion (3%) generates the probe and reference pulses from front and back Fresnel reflections from a CaF_2_ window. The larger portion (97%) goes through a Mach–Zehnder interferometer, which creates two pump pulses with controllable time delay (coherence time, *t*_1_), and a variable translation stage, which delays both pump pulses relative to the probe (population time, *t*_2_). The pulses are directed through the sample plane. The emitted third-order signal and the probe pulse are sent to a diffractive spectrograph (Horiba iHR320), which gets diffracted by a 150 lines/mm grating onto a liquid nitrogen-cooled 2 × 32 MCT array detector (InfraRed Systems Development, Inc., FPAS). Second-order diffraction from the grating provides a higher resolution in the detection frequency ω_3_-axis (0.87 cm^–1^). The *t*_1_ delay was scanned from −0.5 to 15 ps giving a 0.84 cm^–1^ resolution in the excitation frequency, ω_1_.

#### Data Analysis

The FFCFs were extracted from the 2D-IR spectra by using the change in their center line slope.^43^ In short, slices along ω_1_ were taken from the middle of the band, and the minima were plotted as a line. The change in the slope of this line corresponds to the normalized FFCF and fits to the long-time term of the stretched exponential, described in eq 2. We do not report the amplitudes in the main text due to the known differences between the amplitudes extracted from experiments and simulations. Short time scale motions (i.e., less than 0.3 ps) cannot be resolved in our experiments due to our pulse widths (about 0.2 ps fwhm) and motional narrowing effects that blur inertial motions together.

## Supplementary Material



## Notes

Supplementary error analysis of the aCDF and qCDF data, additional MSD, OCF, and CO_2_ angle correlation plots, DVR frequency scan results, and details of spectroscopic map coefficients (PDF)
