# Supplementary material for: Assessing the Effect of Explicit Polarizability on Models of Carbon Dioxide Solvation in Ionic Liquids
Source: ACS Omega. 2025 Nov 11;10(46):56349–63. doi: 10.1021/acsomega.5c08258 (PMC12658653; doi:10.1021/acsomega.5c08258)
Supplement: Supplementary file 1 [file ao5c08258_si_001.pdf]

Supporting Information: Assessing the Effect of Explicit Polarizability on Models of Carbon Dioxide Solvation in Ionic Liquids

Zijian Huo,† Logan E. Smith,† Rowan J. Goudy,† Khady Ndiaye,† Scott Kaiser,† Marie E. Nikolov,‡ Ethan Silva,† Tyler A. Parrack,¶ Sean Garrett-Roe,¶ and Clyde A. Daly Jr.*,†

†*Department of Chemistry, Haverford College, 370 Lancaster Ave., Haverford, Pennsylvania 19041, USA*

‡*Department of Chemistry, Bryn Mawr College, 101 N Merion Ave, Bryn Mawr, PA 19010, USA*

¶*Department of Chemistry, University of Pittsburgh, 219 Parkman Ave., Pittsburgh, PA 15260, USA*

E-mail: cdaly2@haverford.edu

Phone: (610)896-1000

Contents

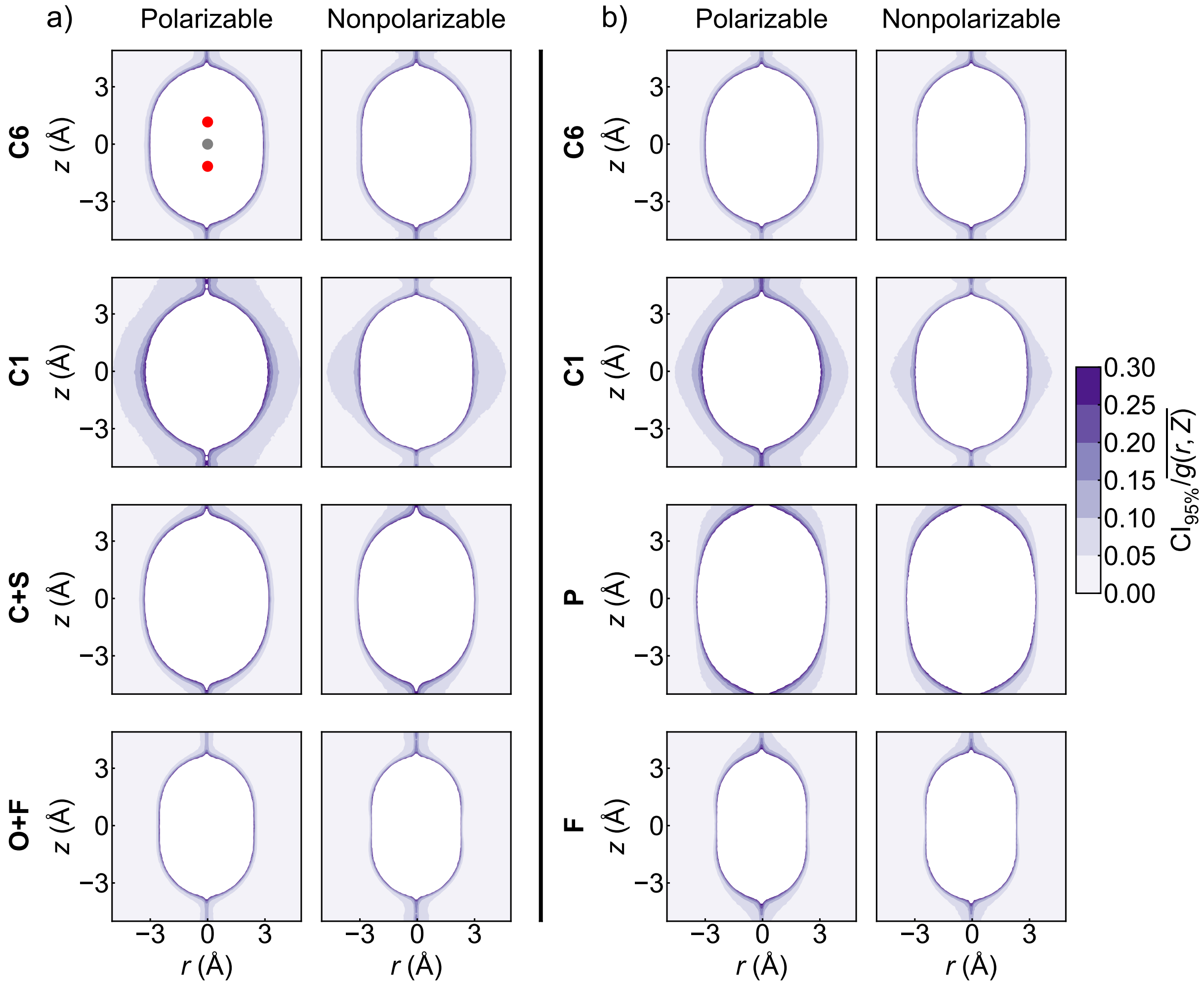

Figure S1: The ratio of 95 % confidence interval to the mean of the atomic density distribution around a single CO_2 molecule in (a) [BMIM$^+$][Tf$_2$N$^-$] and (b) [BMIM$^+$][PF$_6^-$] with polarizable and nonpolarizable force fields.

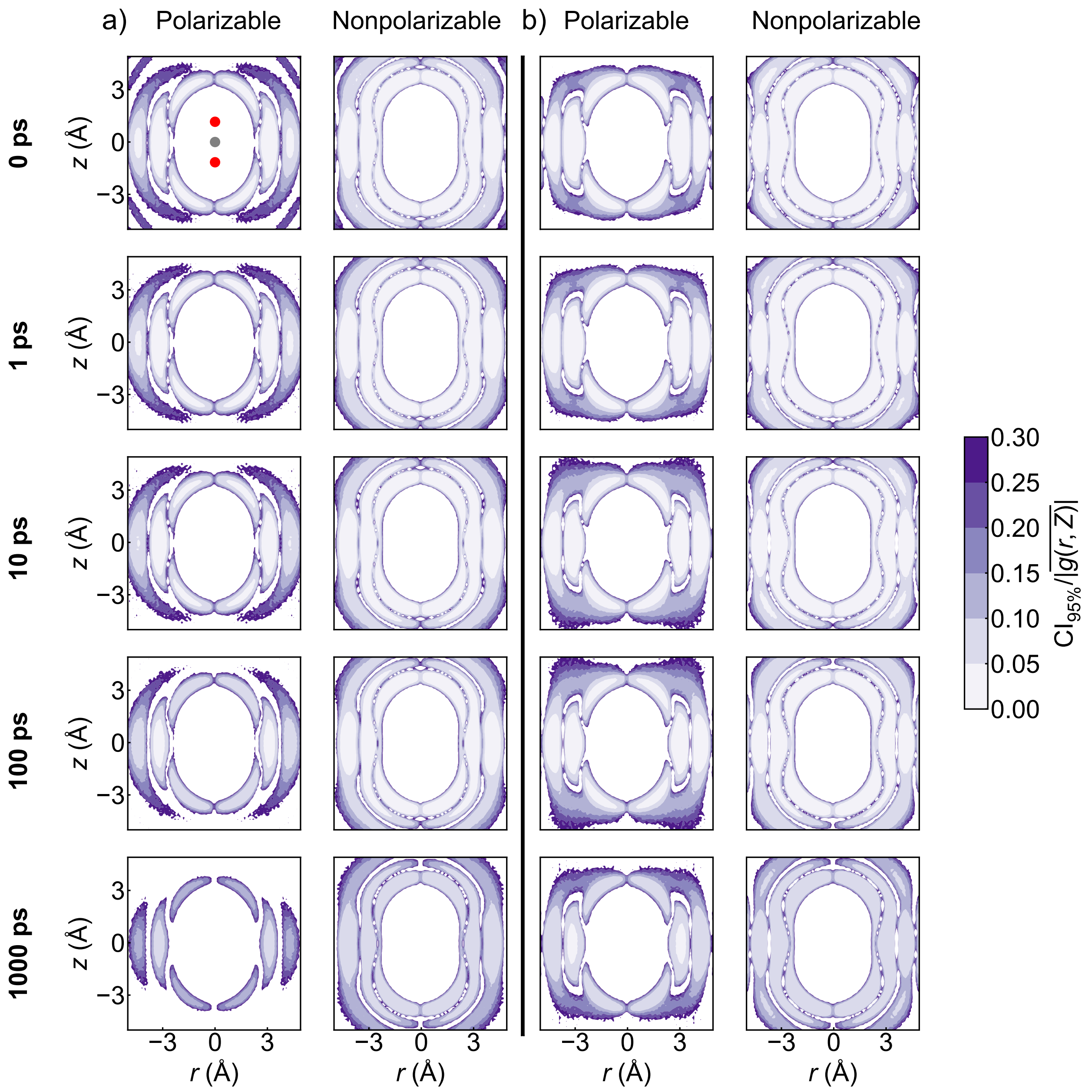

Figure S2: The ratio of 95 % confidence interval to the absolute mean of the charge density of (a) [BMIM$^+$][Tf$_2$N$^-$] and (b) [BMIM$^+$][PF$_6^-$] around a single CO_2 molecule with polarizable and nonpolarizable force fields over time.

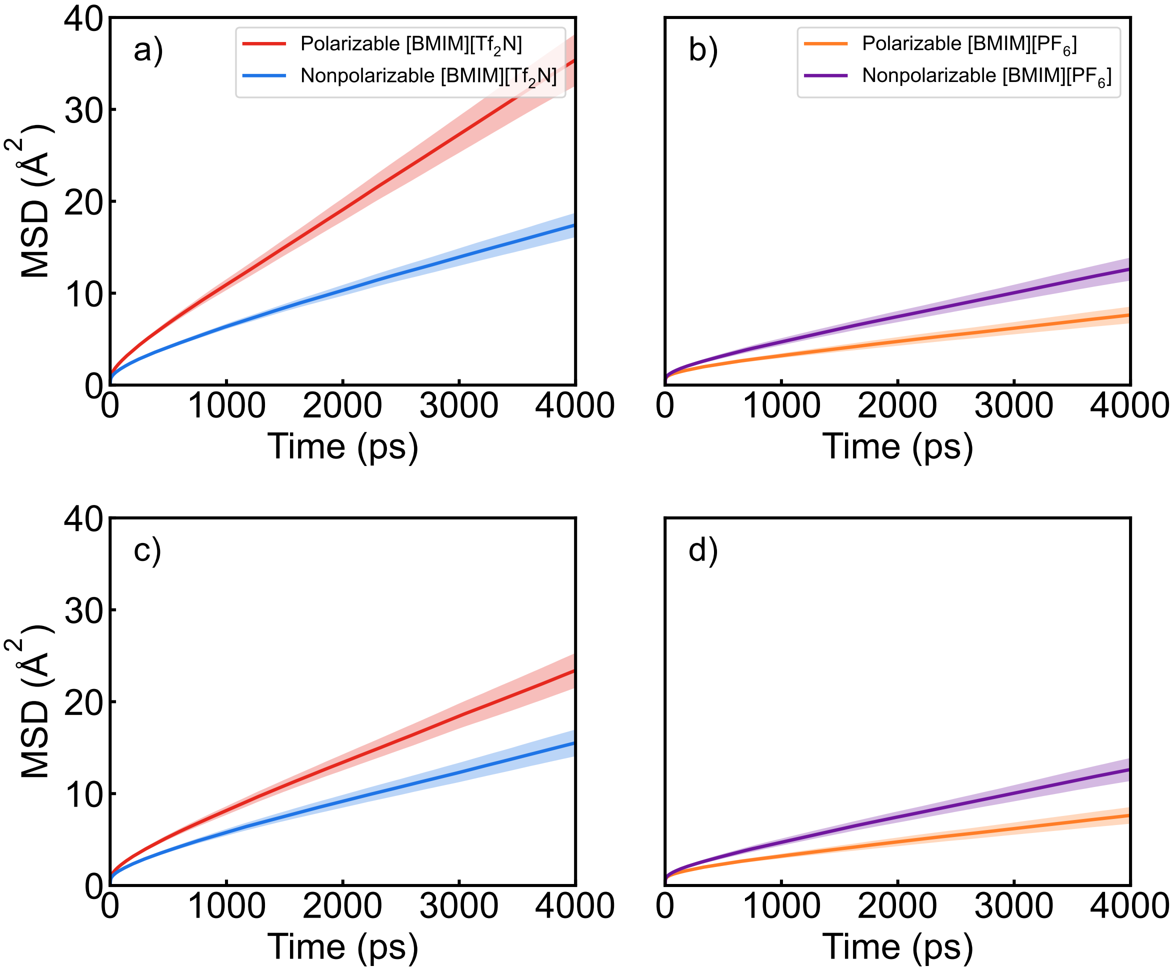

Figure S3: Mean squared displacement of [BMIM$^+$] cations between 0 ns and 4 ns in (a) [BMIM$^+$][Tf_2N^-] and (b) [BMIM$^+$][PF_6^-] with polarizable and nonpolarizable force fields. Mean squared displacement of [Tf_2N^-] and [PF_6^-] anions between 0 ns and 4 ns in (c) [BMIM$^+$][Tf_2N^-] and (d) [BMIM$^+$][PF_6^-] with polarizable and nonpolarizable force fields. The shaded region represents 95% confidence interval of the collected data.

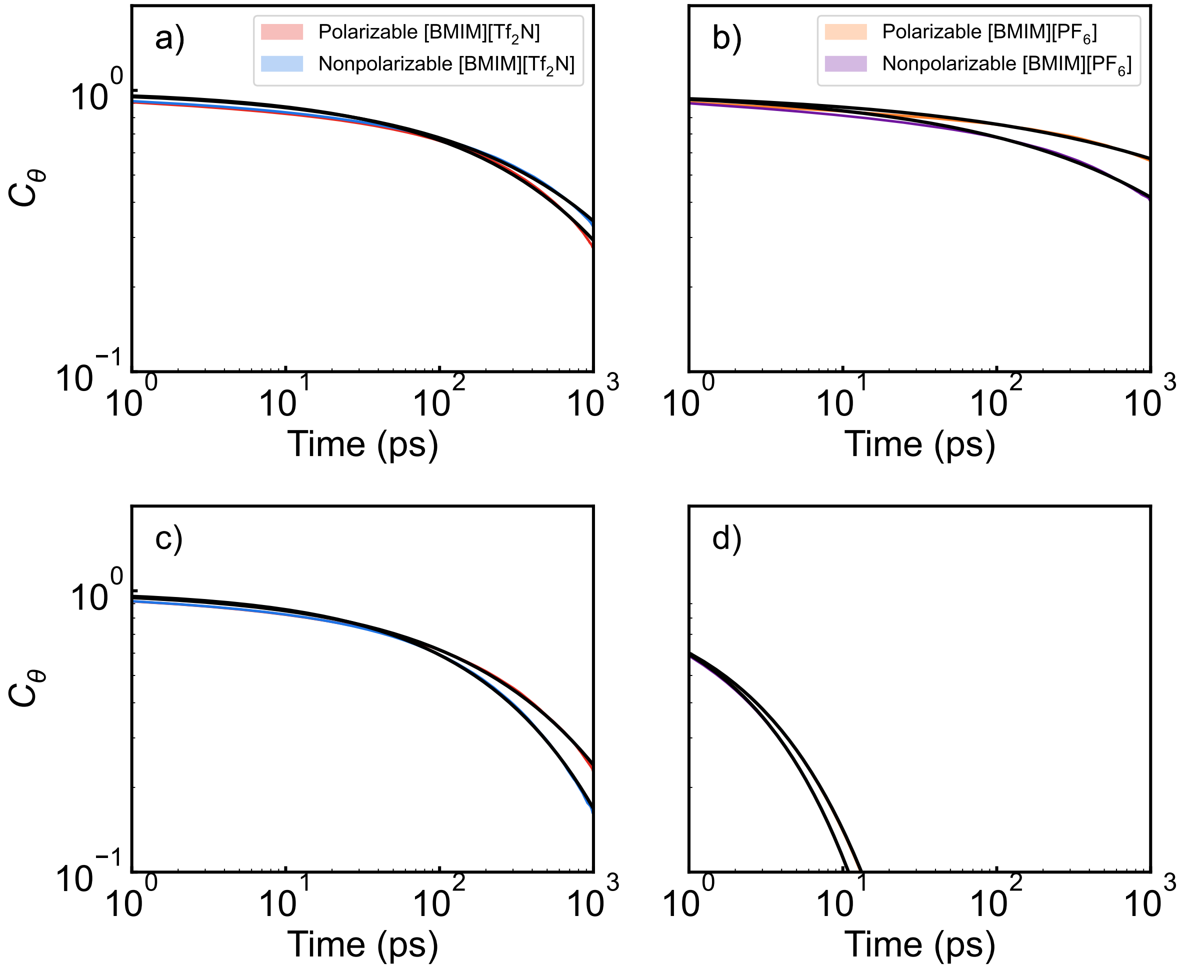

Figure S4: OCFs of [BMIM$^+$] cations calculated from the N1–N11 bond between 1 ps and 1000 ps in (a) [BMIM$^+$][Tf_2N^-] and (b) [BMIM$^+$][PF_6^-] with polarizable and nonpolarizable force fields. OCFs of [Tf_2N^-] and [PF_6^-] anions calculated from the Stf–Stf1 bond and P–F1 bond, respectively, between 1 ps and 1000 ps in (c) [BMIM$^+$][Tf_2N^-] and (d) [BMIM$^+$][PF_6^-] with polarizable and nonpolarizable force fields. The shaded region represents the collected data with the 95 % confidence interval, and the fitted curves are shown in black.

Table S1: Orientational relaxation correlation time of [BMIM$^+$], [Tf_2N^-], and [PF_6^-] in [BMIM$^+$][Tf_2N^-] and [BMIM$^+$][PF_6^-].

**System**	[BMIM$^+$][Tf_2N^-]		[BMIM$^+$][PF_6^-]	
	**Pol**	**Nonpol**	**Pol**	**Nonpol**
τ_θ^+ (ps)	1419	2245	35609	6600
τ_θ^- (ps)	901	553	4.9	4.1

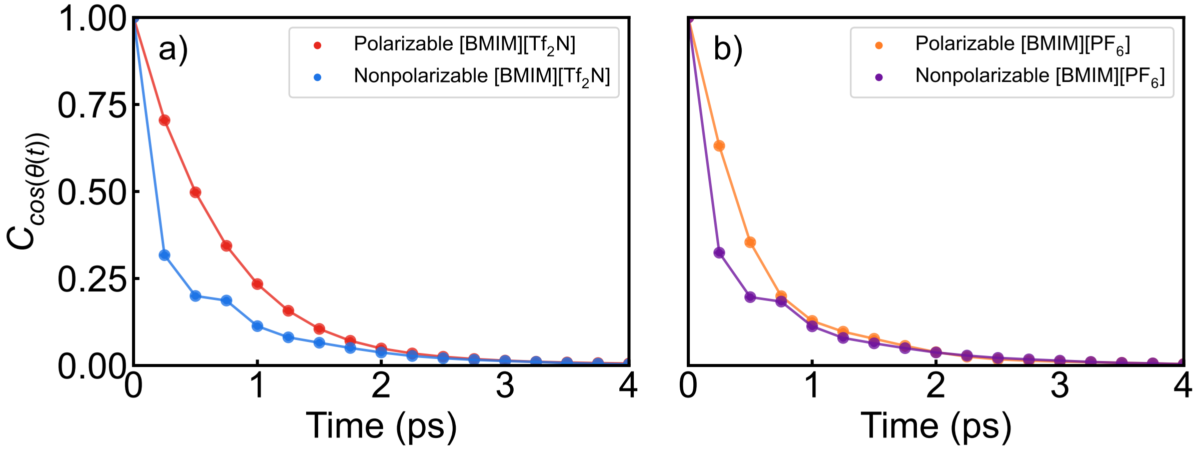

Figure S5: Correlation function for the cosine of the OCO angle of a CO_2 molecule in (a) [BMIM$^+$][Tf$_2$N$^-$] and (b) [BMIM$^+$][PF$_6$$^-$] with polarizable and nonpolarizable force fields. The 95% confidence interval is smaller than the marker size, which is not shown in the plot.

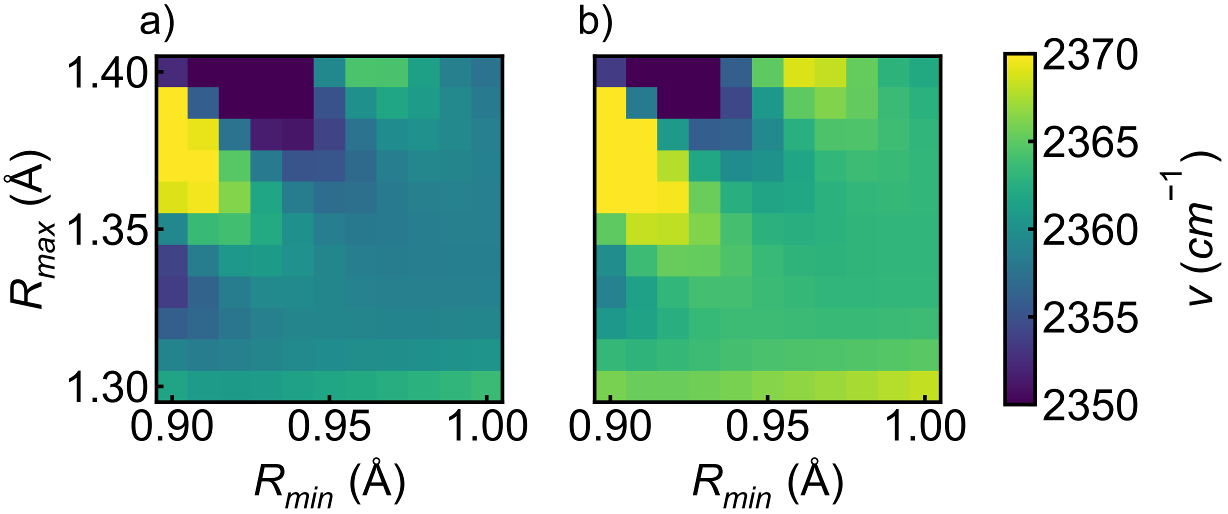

Figure S6: DVR frequency scan for CO_2 asymmetric stretch for randomly selected snapshots of CO_2 and one ion pair of [BMIM$^+$][PF$_6$$^-$] from MD simulations run with a (a) polarizable force field or (b) nonpolarizable force field. In both cases, the frequency is least sensitive to the selection of bond length ranges at higher R_{min} and lower R_{max}. In the end, the values $R_{min} = 0.98$ Å and $R_{max} = 1.35$ Å were selected.

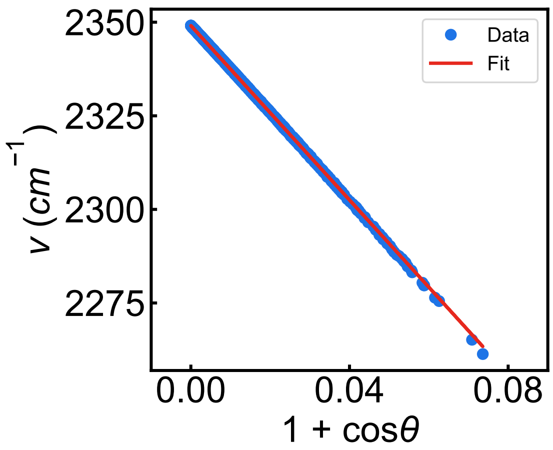

Figure S7: The asymmetric stretch frequency of gas phase CO_2 as a function of the OCO angle. The linear fit was found to be y = -1164.67·x + 2349.10 with $R^2 = 0.9999$.

Table S2: Multi-linear regression vibrational spectroscopic map coefficients for the IL solvent contribution of the non-polarizable force field to the asymmetric vibrational frequencies of CO_2, along with the contributions of each map variable to the overall vibrational frequency. $\langle\Delta\omega\rangle$ represents the average frequency contribution, and $\sigma_{\Delta\omega}$ represents the associated standard deviation.

**Coefficient**	**Term**	**Value**	$\langle\Delta\omega\rangle$, cm^{-1}	$\sigma_{\Delta\omega}$, cm^{-1}
b_1	E_O^{cation}	169 cm^{-1}·au^{-1}	-1.23	0.57
b_2	E_O^{anion}	417 cm^{-1}·au^{-1}	-0.79	1.56
c_1	U_O	3.23 cm^{-1}·kJ^{-1}·mol	-7.02	1.81
c_2	U_C	-2.28 cm^{-1}·kJ^{-1}·mol	5.15	1.55

Table S3: Multi-linear regression vibrational spectroscopic map coefficients for the IL solvent contribution of the polarizable force field to the asymmetric vibrational frequencies of CO_2, along with the contributions of each map variable to the overall vibrational frequency. $\langle\Delta\omega\rangle$ represents the average frequency contribution, and $\sigma_{\Delta\omega}$ represents the associated standard deviation.

**Coefficient**	**Term**	**Value**, cm^{-1}·kJ^{-1}·mol	$\langle\Delta\omega\rangle$, cm^{-1}	$\sigma_{\Delta\omega}$, cm^{-1}
a_{11}	$E^{+}_{O_{\mathrm{elec}}}$	-2.65	-3.95	5.78
a_{12}	$E^{-}_{O_{\mathrm{elec}}}$	0.950	2.85	1.23
a_{21}	$E^{+}_{C_{\mathrm{elec}}}$	-1.80	-6.36	2.36
a_{22}	$E^{-}_{C_{\mathrm{elec}}}$	0.00487	0.00	0.01
b_{11}	$E^{+}_{O_{\mathrm{exch}}}$	1.67	11.58	8.89
b_{12}	$E^{-}_{O_{\mathrm{exch}}}$	0.626	2.99	2.14
b_{21}	$E^{+}_{C_{\mathrm{exch}}}$	0.323	0.83	0.80
b_{22}	$E^{-}_{C_{\mathrm{exch}}}$	-0.0358	-0.18	0.17
c_{11}	$E^{+}_{O_{\mathrm{disp}}}$	1.51	-9.76	4.19
c_{12}	$E^{-}_{O_{\mathrm{disp}}}$	0.326	-2.29	1.04
c_{21}	$E^{+}_{C_{\mathrm{disp}}}$	-1.01	4.83	2.15
c_{22}	$E^{-}_{C_{\mathrm{disp}}}$	0.180	-1.28	0.65
d_{11}	$E^{+}_{O_{\delta\mathrm{HF}}}$	3.43	1.25	1.34
d_{12}	$E^{-}_{O_{\delta\mathrm{HF}}}$	-3.37	-0.89	0.69
d_{21}	$E^{+}_{C_{\delta\mathrm{HF}}}$	0.400	0.05	0.07
d_{22}	$E^{-}_{C_{\delta\mathrm{HF}}}$	-5.10	-1.55	1.59
